# Incorporating Time Delays in Process Hitting Framework for Dynamical Modeling of Large Biological Regulatory Networks

**DOI:** 10.3389/fphys.2019.00090

**Published:** 2019-02-15

**Authors:** Iftikhar Ali Sheikh, Jamil Ahmad, Morgan Magnin, Olivier Roux

**Affiliations:** ^1^Research Centre for Modeling and Simulation, National University of Sciences and Technology, Islamabad, Pakistan; ^2^Department of Computer Science and Information Technology, University of Malakand, Chakdara, Pakistan; ^3^Laboratory of Digital Sciences of Nantes (LS2N), UMR CNRS 6004, Ecole Centrale de Nantes, Nantes, France

**Keywords:** process hitting, logical modeling, René Thomas framework, kinetic logic, time delays, hybrid modeling, systems biology, biological regulatory networks

## Abstract

Modeling and simulation of molecular systems helps in understanding the behavioral mechanism of biological regulation. Time delays in production and degradation of expressions are important parameters in biological regulation. Constraints on time delays provide insight into the dynamical behavior of a Biological Regulatory Network (BRN). A recently introduced Process Hitting (PH) Framework has been found efficient in static analysis of large BRNs, however, it lacks the inference of time delays and thus determination of their constraints associated with the evolution of the expression levels of biological entities of BRN is not possible. In this paper we propose a *Hybrid Process Hitting* scheme for introducing time delays in Process Hitting Framework for dynamical modeling and analysis of Large Biological Regulatory Networks. It provides valuable insights into the time delays corresponding to the changes in the expression levels of biological entities thus possibly helping in identification of therapeutic targets. The proposed framework is applied to a well-known BRNs of *Bacteriophage* λ and ERBB Receptor-regulated G1/S transition involved in the breast cancer to demonstrate the viability of our approach. Using the proposed approach, we are able to perform goal-oriented reduction of the BRN and also determine the constraints on time delays characterizing the evolution (dynamics) of the reduced BRN.

## 1. Introduction

Biological Regulatory Networks (BRNs) are used to describe almost all biological functions to cover the interactions taking place in various chemical reactions in living organisms. The nature of these interactions is continuous and stochastic in nature and the rate of change in the underlying dynamics of these interactions is variable. A large number of formal approaches have been devised to model the topology of BRNs and to analyse their dynamical behaviors (De Jong, 2002; Sheikh et al., 2016). BRNs are traditionally modeled by differential equations but they are non-linear and thus difficult to solve analytically. Moreover, the available experimental data is mostly qualitative in nature and do not aid in precise determination of quantitative parameters for the differential equations. A powerful tool involving Lie algebra has been reported to solve a large class of non-linear epidemic spreading systems analytically (Shang, 2012, 2015a), however it has not been applied to the dynamical modeling of BRNs. René Thomas Discrete Modeling technique (Thomas, 1978) proposed a qualitative method for representing the change in expression level of a gene or biological entity with a logical function having discrete values. The information on whether the gene expression would increase or decay is contained in the *K-parameters* associated with each entity of the BRN. The values of these *parameters* determine the dynamical behavior of the system that is represented by a State Transition Graph (STG). The state graph grows exponentially with the increase in the number of entities thus it becomes difficult to analyse medium or large scale BRNs (containing more than 10 or more genes). Moreover, this approach ignores the time taken for the gene to reach the expression level required for production or degradation and therefore, uses an asynchronous evolution of the dynamics of the system. Hybrid Modeling Technique (Ahmad et al., 2006) addressed this shortcoming by associating time delays in BRNs by considering the change in qualitative levels as piece-wise linear functions. This made it possible to perform model checking and obtain constraints in terms of production and/or degradation delays. The hybrid approach works well for BRNs consisting up to 7–8 genes, however, it is unable to compute the time delays for larger networks due to the state space explosion.

A Process Hitting framework for analysis of large BRNs has been proposed (Paulevé et al., 2011; Folschette et al., 2012) which can handle networks comprising of thousands of genes. The very high scalability of this approach is due to the following factors:

- It takes into consideration only the most permissible (generalized) dynamics possible in the interaction graph of a BRN instead of dealing with the whole state space.
- The generalized dynamics is refined with the help of cooperativity between two or more genes, which have a combined influence on any other gene in the network.

The Process Hitting approach is thus able to quickly perform static analysis like determination of stable states, successive reachability and inferring the *K-parameters* of the BRN. This technique is based on Stochastic π-Calculus and allows for synthesis of temporal and stochastic parameters, which enables the simulation of dynamical behavior to some extent. However, there is no mechanism for incorporating the time delays in the proposed framework. Lately, time parameters have been introduced into Process Hitting with classes of priorities (Folschette et al., 2015); however, it still does not infer time delays of the interactions.

The Process Hitting is also able to perform goal-oriented reduction of the large BRNs by taking into consideration the minimal traces that are necessary to achieve the desired reachability goal. It applies the cutsets to preserve the minimal traces through Gene Knock-out/in/down technique (Paulevé et al., 2013).

Previously, enhanced modeling of BRNs based on timed automata has been performed in Siebert and Bockmayr (2008) which introduced time delays in the René Thomas Discrete Modelling Framework and it is possible to simulate the dynamics of BRN. However, this approach does not infer the constraints on time delays rather the numerical values have to be adjusted manually to obtain a certain behavior. Moreover, it is also limited to small BRNs as it introduces intermediate states, which results in an even greater number of possible states in the state transition graph.

Recently, new methodology has been proposed for the inference of BRNs through a time extension of Automata Networks using the time series data and known influences among the genes (Ben Abdallah et al., 2017). This modeling approach requires the observed experimental time series data for deducing the BRN model which satisfies the dynamical behavior depicted by the observed data.

Our approach is to extend the Process Hitting Framework by introducing *Time Delays* in it so that it becomes possible to determine the constraints on the activation and degradation delays associated with the evolution of a gene in the dynamical model of the BRN. In order to achieve it, we represent the biological entities of the BRN with Biological Linear Hybrid Automata (Bio-LHA) (Ahmad et al., 2009; Aslam et al., 2014) and enrich them with time delays while the logical rules for gene activation or degradation are provided by the cooperative hits as determined by Process Hitting. Since PH only takes into consideration the permissible (possible) dynamics of the BRN as given in its Interaction Graph (IG), therefore, we have a reduced STG to be represented by the Hybrid Automata.

The proposed approach, *Hybrid Process Hitting*, has been applied to a toy BRN to fully explain the steps involved and subsequently applied to the biologically well-known network of *Bacteriophage* λ and ERBB Receptor-regulated G1/S transition involved in the breast cancer to successfully determine the constraints on Time Delays in less computational time. Moreover, due to its reduced complexity, the proposed approach would be able to handle BRNs with more number of genes as compared to the Hybrid Modeling approach that takes into account the complete STG while determining the constraints on Time Delays.

The rest of the paper is organized as follows: section 2 discusses the Process Hitting Framework while the basics of René Thomas Discrete Modelling Framework and Hybrid Modeling are described in section 3. The proposed methodology for incorporation of Time Delays in Process Hitting Framework is given in section 4 and the proposed *Hybrid Process Hitting* approach is applied to the BRNs of *Bacteriophage* λ and ERBB-receptor regulated G1/S transition in sections 5, 6 followed by concluding remarks and future work in sections 7, 8, respectively. A glossary of mathematical notations used in the definitions in this paper is provided as supplementary file (Data Sheet 2).

## 2. Process Hitting (PH) Framework

This section describes the Process Hitting Framework which is used for the static analysis of large scale biological regulatory networks (Paulevé et al., 2011; Folschette et al., 2012) and Goal-oriented model reduction.

### 2.1. Biological Regulatory Networks (BRNs)

BRNs are used to model the interactions between various biological entities (Proteins or RNA). Quite a few complex processes are involved in the regulations between them, however, these are simplified to only two actions, namely, activation and inhibition (Thomas and d'Ari, 1990). BRNs are often described as an Interaction Graph in which genes or other biological entities are shown as nodes and their activating or inhibiting influences on the other elements are represented by positive or negative edges, respectively. The simple representation of BRN was extended to automatically derive its behavioral model by using model-checking tools (Bernot et al., 2007). The discrete levels are defined for a particular concentration level of a gene after which its influence on other entities in the BRN changes. The *activating* (resp. *inhibiting*) influence of a gene is exerted once it is *above* (resp. *below*) the threshold level.

**Definition 1. (Interaction Graph)**. *An Interaction Graph* *IG* = (*N, E*) *of a Biological Regulatory Network is defined where* *N* *is the set of all nodes and* *E* *is the set of all directed edges in which an edge from node* *p* *to* *q* *is* $e=\left(p\overset{t,s}{\underset{}{\to }}q\right)$ *and is labelled by the pair* (*t, s*) *such that* *t* *is a positive integer and represents the qualitative threshold level required for interaction, and* *s* ∈ {+, −} *describes the type of interaction, i.e., “+” for activation and “*−”* for inhibition*.

The interaction graph described above is quite similar with the discrete version of Deffuant model in opinion evolution in a network (Shang, 2015b).

**Definition 2. (Effective Levels)**. *For a regulation* $p\overset{t,s}{\underset{}{\to }}q$ *in a BRN and* *s* *being* + *(resp*. −*), the effective levels* $LEV_{p}^{+}$ *are those threshold levels* {*t*; *l*_*p*_} (*resp*.{0;*t*−1}) *of gene p at and above (resp. below) which it will activate (resp. inhibit) gene* *q, where* *l*_*p*_ *is the highest threshold level of gene* *p. Conversely, effective levels* $LEV_{p}^{-}$ *would be* {0;*t*−1}(*resp*.{*t*; *l*_*p*_}) *for which gene* *q* *would be activated by gene* *p*.

**Figure 1:**
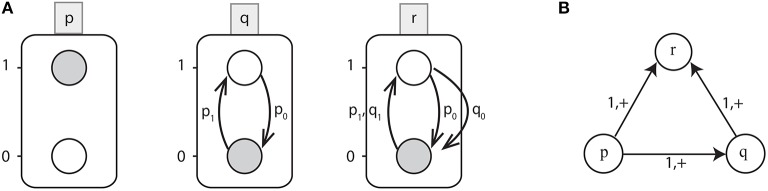
**(A)** An example of Automata Network (AN). Labeled boxes represent the Genes (*automaton*) whereas the circles represent the Levels (local states) within an *automata* and labeled with numbers, e.g., *p*_0_ is the local state labeled *0* in the box *p*. Also the local transitions (e.g., $q_{0}\overset{\left\{p_{1}\right\}}{\underset{}{\to }}q_{1}$) are represented by arrows labeled by their necessary conditions. The network state 〈*p*_1_, *q*_0_, *r*_0_〉 is depicted by grayed local states. **(B)** Interaction graph of a toy BRN.

**Example 1**. In the BRN shown in Figure 1B, the edge $p\overset{1,+}{\underset{}{\to }}q$ depicts the activating influence of gene *p* on gene *q* at threshold level 1. So effective levels $LEV_{p}^{+}$ are {1;1} and $LEV_{p}^{-}$ are {0;0} where gene *p* would be inhibiting gene *q*. Similarly, for some other interaction $p\overset{2,-}{\underset{}{\to }}q$ with *l*_*p*_ = 3, the effective levels $LEV_{p}^{+}$ are {0;1} and $LEV_{p}^{-}$ are {2;3}.

### 2.2. Automata Networks

Starting from any arbitrary state of the system, Process Hitting takes into account all the possible evolutions (Generalized Dynamics) of the BRN and keeps evolving the network till it reaches a steady state where no more interactions can take place. In Process Hitting Framework, each gene is represented as *automata* and its current level as *local state*. A Process Hitting state is formed by gathering one *local state* of each *automata* present in the BRN. A *local state* *i* of a *automata* *p* is denoted as *p*_*i*_. At any given time for each automaton only one local state is present which represents the current level of activity of that gene. Accordingly, the set of all local states is the global state of the BRN. The local transition is the local possible evolutions inside an automaton. The transition $p_{i}\overset{l}{\underset{}{\to }}p_{j}$ is the change of local state *p*_*i*_ to local state *p*_*j*_ made possible by the presence of a set *l* of local states belonging to other automata which should be active in the current global state. Thus, all local states in *l* cooperate to switch the active local state of *p* from *p*_*i*_ to *p*_*j*_. It is noted that *l* contains at most one local state of another automaton and none from automaton *p*; it can also be empty. Automata Network is formally defined as:

**Definition 3. (Automata Network)**. *An Automata Network (AN) is a triple* (Σ,L,H) *where:*

- Σ = {*p, q*, …} *is the finite set of automata*,
- *L* = Π_*p* ∈ Σ_*L*_*p*_ *is the finite set of global states with* *L*_*p*_ = {*p*_0_, …, *p*_*l*_*p*__} *the finite set of local states of automata* *p* ∈ Σ *and* *l*_*p*_ *a positive integer. Also* *p* ≠ *q* ⇒ ∀(*p*_*i*_, *q*_*j*_) ∈ *L*_*p*_ × *L*_*q*_, *p*_*i*_ ≠ *q*_*j*_*, and* **LS** = ∪_*p* ∈ Σ_*L*_*p*_ *denotes the set of all the local states*.
- *for each* $p\in \Sigma ,H_{p}=\left\{p_{i}\overset{l}{\underset{}{\to }}p_{j}\in L_{p}\times ℘\left(\text{LS}\backslash L_{p}\right)\times L_{p}|p_{i}\neq p_{j}\right\}$*, is the set of local transitions on automata* *p;* $H=\cup_{p\;\in \Sigma }H_{p}$ *is the set of all local transitions in the network*.

**Definition 4. (Step)**. *For an AN* (Σ,L,H)*, a step* θ⊆H *is a subset of local transitions* H *where, for each automaton* *a* ∈ Σ*, there is at most one local change in a*.

For any local transition $\theta =p_{i}\overset{\ell }{\underset{}{\to }}p_{j}$, *p*_*i*_ is the origin, ℓ is the condition and *p*_*j*_ is the destination and, respectively, noted as ^•^*θ*, *cond(θ)* and *θ*^•^.

**Definition 5. (Trace)**. *For an AN* (Σ,L,H) *and a state* *l* ∈ *L, a trace* π *is a sequence of successive steps* *θ* *which leads to a defined goal state* *l*_*T*_. *The trace π is denoted as* *θ*_1_::*θ*_2_::… .

Given an Automata Network (Σ,L,H) and a state *l* ∈ *L*, the goal state *l*_*T*_ ∈ *L* is reachable from *i* if there exists a trace *π* with ^•^*π* ⊆ *l* and *l_T_* ∈ π^•^.

**Example 2**. Figure 1A shows the Automata Network of a toy BRN depicted in Figure 1B where:

- Σ = {*p, q, r*},
- *L*_*p*_ = {*p*_0_, *p*_1_}, *L*_*q*_ = {*q*_0_, *q*_1_}, *L*_*r*_ = {*r*_0_, *r*_1_} and
- the set of transitions as:

$$\begin{array}{l} H=\{q_{0}\overset{\left\{p_{1}\right\}}{\to }q_{1},\;q_{1}\overset{\left\{p_{0}\right\}}{\to }q_{0},\;r_{0}\overset{\left\{p_{1},q_{1}\right\}}{\to }r_{1},\;r_{1}\overset{\left\{p_{0}\right\}}{\to }r_{0},\\ \;r_{1}\overset{\left\{q_{0}\right\}}{\to }r_{0}\}. \end{array}$$

Starting from the state *l* = 〈*p*_1_, *q*_0_, *r*_0_〉, only one transition is playable: $\theta =q_{0}\overset{\left\{p_{1}\right\}}{\underset{}{\to }}q_{1}$ which updates the automaton state to *l* · θ = 〈*p*_1_, *q*_1_, *r*_0_〉. From this state, the next possible transition is $r_{0}\overset{\left\{p_{1},q_{1}\right\}}{\underset{}{\to }}r_{1}$ which leads to the state 〈*p*_1_, *q*_1_, *r*_1_〉. Thus, from the state *l* = 〈*p*_1_, *q*_0_, *r*_0_〉 to 〈*p*_1_, *q*_1_, *r*_1_〉, the trace π is given by: $\pi =\left\{q_{0}\overset{\left\{p_{1}\right\}}{\underset{}{\to }}q_{1}\right\}::\left\{r_{0}\overset{\left\{p_{1},q_{1}\right\}}{\underset{}{\to }}r_{1}\right\}.$

### 2.3. Goal Oriented Reduction and Cutsets

In the Automata Networks representing the BRNs, a goal is typically the *activation (resp. inhibition)* of some gene or transcription factor or kinase, etc. From an initial given state for each entity in BRN, the goal is reachable if a sequence of steps is present which leads to the state which contains the goal (Paulevé, 2018). For example, the goal in a signaling network is usually the activation of the downstream transcription factor starting from the initial inactive state.

A *minimal trace* corresponds to the path or sequence of steps (containing no cycle) starting from the initial state to the defined goal state containing the gene activation / inhibition. In a cycle, the initial global state is visited twice, i.e., the path leads back to its starting point. There can be multiple *traces* which satisfy the goal reachability in the network. So, the goal oriented reduction aims to preserve all *minimal traces* to the defined goal.

For a global AN (Σ,L,H), an initial state *l* ∈ *L* and a reachability goal state *l*_*T*_, the goal oriented reduction identifies a subset of local transitions H that are sufficient for producing all the minimal traces leading to *l*_*T*_ from *l*.

#### 2.3.1. Cutsets for Goal Reachability

**Figure 2:**
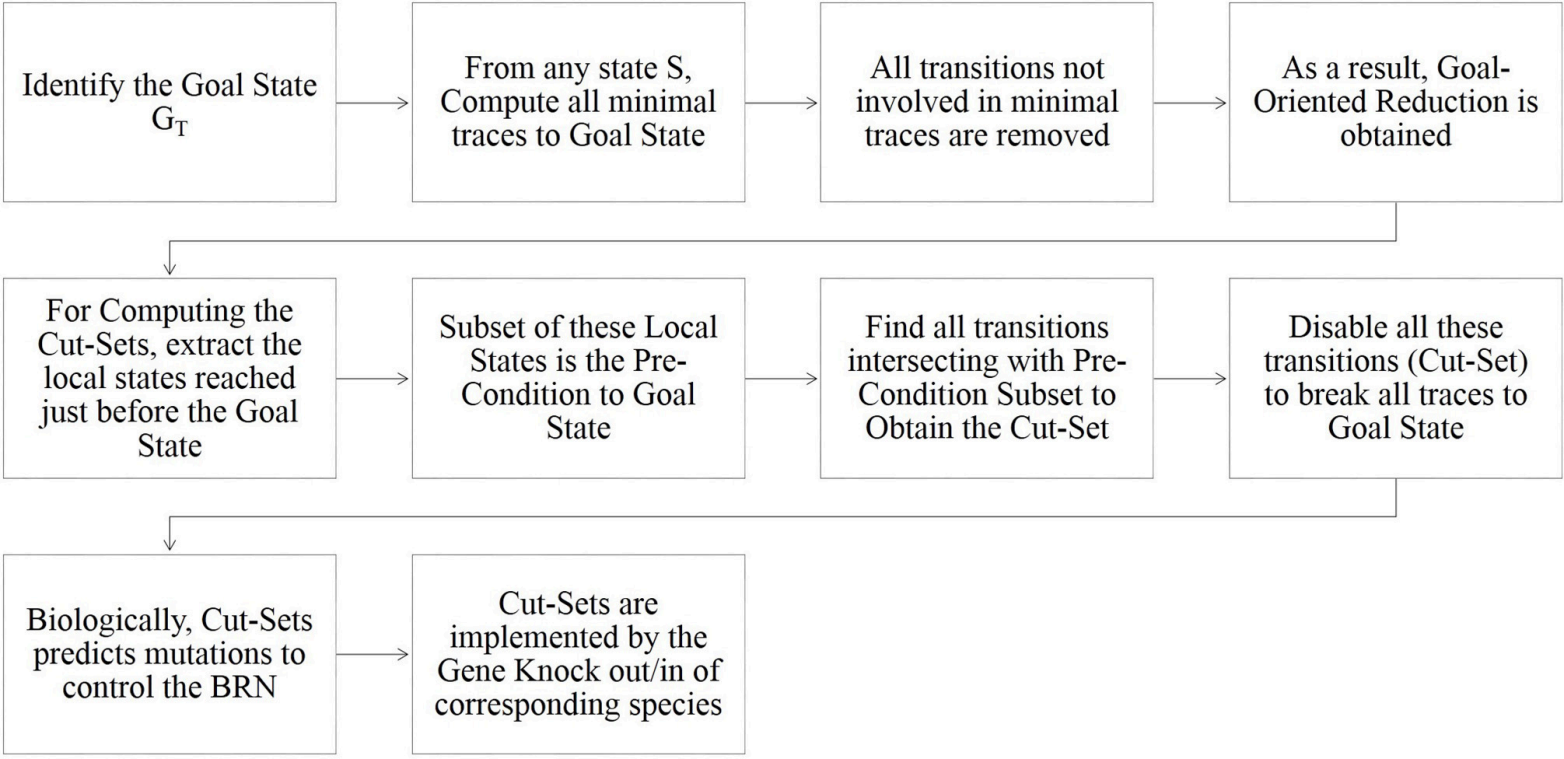
Explanatory image of the algorithm for computation of Goal-Oriented Reachability and Cut-Sets in Process Hitting (Paulevé, 2018).

*Cutsets* are the sets of local states such that each trace reaching the goal includes a transition involving one of these local states. Also, these sets contain the necessary processes which when disabled would prevent the considered reachability goal. Hence, disabling of all transitions having precondition intersecting with cutset will remove all the traces leading to the goal. The algorithm for calculating the Cutset is described in detail in Paulevé et al. (2013) alongwith the complete methodology. An explanatory image describing the algorithm is given in Figure 2.

The *Cutsets* provide information on potential therapeutic targets if the considered reachability goal represents a diseased condition by preventing all the local states of a cutset to act using other Gene Knock-out/in/down techniques (Paulevé et al., 2013).

### 2.4. Regulation Conditions for Cooperative Hits

The combined effect or influence of multiple genes in a BRN is governed by logical functions (Richard et al., 2006; Bernot et al., 2008) unless there are biological observations which provide evidence that the genes are exerting an influence other than that given by the logical function. In that case the biological observation take precedence over the logical function.

**Figure 3:**
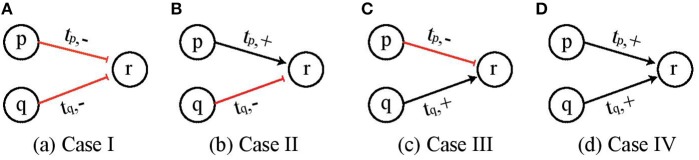
Four possible cases of cooperation of two genes *p* and *q* to influence a third gene *r*. **(A)** Both *p* and *q* are inhibitors and would upregulate gene *r* when both are below threshold value *t*_*p*_ and *t*_*q*_, respectively. **(B–D)** Other possible combinations of gene *p* and *q*.

Here we give all the possible cases for two genes *p* and *q* to cooperate and exert influence on a third gene *r* at threshold levels *t*_*p*_ and *t*_*q*_, respectively, and define the rules for logical functions for activation (upregulation) of gene *r* when both *p* and *q* are acting simultaneously. Figure 3 depicts the four cases which cover all possible combinations of two entities which are ¬*p* ∧ ¬*q*, ¬*p* ∧ *q*, *p* ∧ ¬*q* and *p* ∧ *q*.

**Definition 6. (Regulation Condition for Individual Hits)**. *The regulation condition* *Cond*_*ACT*_ *(resp*. *Cond*_*INH*_*) for the individual hit contained in the set* H *by a gene* *p* *having interaction with gene* *r* *as* $\left(p\overset{t_{p},s_{p}}{\underset{}{\to }}r\right)$ *is defined as:*

$$\begin{array}{l} cond_{ACT}=\{\begin{array}{ll} p_{L}, & \;s_{p}=\{-\}\\ p_{H}, & \;s_{p}=\{+\} \end{array}\text{ and}\\ cond_{INH}=\{\begin{array}{ll} p_{H}, & \;s_{p}=\{-\}\\ p_{L}, & \;s_{p}=\{+\} \end{array} \end{array}$$

Now we consider the combined influence of two genes *p* and *q* on one single entity *r* and define the rule for this cooperative interaction. If both the individual influencers *p* and *q* are the “inhibitors” of target gene *r*, then it will be *activated* when individually both *p* and *q* are at *p*_*L*_ and *q*_*L*_ represented by *pq*_*LL*_. For all other combinations of *p* and *q* given by *pq*_*LH*_, *pq*_*HL*_, *pq*_*HH*_, the target gene *r* would be *inhibited*.

**Definition 7. (Regulation Condition for Cooperative Hits)**. *The regulation condition* *Cond*_*ACT*_ *(resp*. *Cond*_*INH*_*) for the cooperative hit* H *by two genes* *p* *and* *q* *having their individual interactions with gene* *r* *as* $\left(p\overset{t_{p},s_{p}}{\underset{}{\to }}r\right)$ *and* $\left(q\overset{t_{q},s_{q}}{\underset{}{\to }}r\right)$ *respectively is defined as:*

$$cond_{ACT}=\{\begin{array}{cc} pq_{LL}, & \;s_{p}=\{-\},s_{q}=\{-\}\\ pq_{LH}, & \;s_{p}=\{-\},s_{q}=\{+\}\\ pq_{HL}, & \;s_{p}=\{+\},s_{q}=\{-\}\\ pq_{HH}, & \;s_{p}=\{+\},s_{q}=\{+\} \end{array}$$

and

$$cond_{INH}=\{\begin{array}{ll} pq_{LH},pq_{HL},pq_{HH}, & \;s_{p}=\{-\},s_{q}=\{-\}\\ pq_{LL},pq_{HL},pq_{HH}, & \;s_{p}=\{-\},s_{q}=\{+\}\\ pq_{LL},pq_{LH},pq_{HH}, & \;s_{p}=\{+\},s_{q}=\{-\}\\ pq_{LL},pq_{LH},pq_{HL}, & \;s_{p}=\{+\},s_{q}=\{+\} \end{array}$$

We see that for *Case I* (¬*p* ∧ ¬*q*) shown in Figure 3A, the logical AND function implies that *q* would be upregulated when both *p* and *q* are below threshold *t*_*p*_ and *t*_*q*_, respectively, which is represented by *pq*_*LL*_ and it would be downregulated for all other values, i.e., *pq*_*LH*_, *pq*_*HL*_, and *pq*_*HH*_. Once the appropriate regulation condition has been obtained then the corresponding effective levels are determined in a straight forward manner. For *Cond*_*ACT*_ = *pq*_*LL*_ the effective levels are found to be $LEV_{p}^{-}=\left\{0;t_{p}-1\right\}$ and $LEV_{q}^{-}=\left\{0;t_{q}-1\right\}$. Similarly, the conditions and effective levels for *activation* (*resp. inhibition*) for *Cases II, III* and *IV* are given in Table 1.

**Table 1:** The regulation conditions and effective levels for *Up* (resp. *Down*) Regulation by two genes *p* and *q* having a combined influence on another gene in a BRN are governed by the logical function.

**Cases**	**Logical function**	**Activation**		**Inhibition**	
		***Cond*_*ACT*_**	**Effective levels**	***Cond*_*INH*_**	**Effective levels**
I	¬*p* ∧ ¬*q*	*pq*_*LL*_	$LEV_{p}^{-}=\left\{0;t_{p}-1\right\}$ $LEV_{q}^{-}=\left\{0;t_{q}-1\right\}$	*pq*_*LH*_	$LEV_{p}^{-}=\left\{0;t_{p}-1\right\}$ $LEV_{q}^{+}=\left\{t_{q};l_{q}\right\}$ $LEV_{p}^{+}=\left\{t_{p};l_{p}\right\}$
				*pq*_*HL*_	$LEV_{q}^{-}=\left\{0;t_{q}-1\right\}$ $LEV_{p}^{+}=\left\{t_{p};l_{p}\right\}$
				*pq*_*HH*_	$LEV_{q}^{+}=\left\{t_{q};l_{q}\right\}$
II	¬*p* ∧ *q*	*pq*_*LH*_	$LEV_{p}^{-}=\left\{0;t_{p}-1\right\}$ $LEV_{q}^{+}=\left\{t_{q};l_{q}\right\}$	*pq*_*LL*_	$LEV_{p}^{-}=\left\{0;t_{p}-1\right\}$ $LEV_{q}^{-}=\left\{0;t_{q}-1\right\}$ $LEV_{p}^{+}=\left\{t_{p};l_{p}\right\}$
				*pq*_*HL*_	$LEV_{q}^{-}=\left\{0;t_{q}-1\right\}$ $LEV_{p}^{+}=\left\{t_{p};l_{p}\right\}$
				*pq*_*HH*_	$LEV_{q}^{+}=\left\{t_{q};l_{q}\right\}$
III	*p* ∧ ¬*q*	*pq*_*LH*_	$LEV_{p}^{+}=\left\{t_{p};l_{p}\right\}$ $LEV_{q}^{-}=\left\{0;t_{q}-1\right\}$	*pq*_*LL*_	$LEV_{p}^{-}=\left\{0;t_{p}-1\right\}$ $LEV_{q}^{-}=\left\{0;t_{q}-1\right\}$ $LEV_{p}^{-}=\left\{0;t_{p}-1\right\}$
				*pq*_*HL*_	$LEV_{q}^{+}=\left\{t_{q};l_{q}\right\}$ $LEV_{p}^{+}=\left\{t_{p};l_{p}\right\}$
				*pq*_*HH*_	$LEV_{q}^{+}=\left\{t_{q};l_{q}\right\}$
IV	*p* ∧ *q*	*pq*_*HH*_	$LEV_{p}^{+}=\left\{t_{p};l_{p}\right\}$ $LEV_{q}^{+}=\left\{t_{q};l_{q}\right\}$	*pq*_*LL*_	$LEV_{p}^{-}=\left\{0;t_{p}-1\right\}$ $LEV_{q}^{-}=\left\{0;t_{q}-1\right\}$ $LEV_{p}^{-}=\left\{0;t_{p}-1\right\}$
				*pq*_*LH*_	$LEV_{q}^{+}=\left\{t_{q};l_{q}\right\}$ $LEV_{p}^{+}=\left\{t_{p};l_{p}\right\}$
				*pq*_*HL*_	$LEV_{q}^{-}=\left\{0;t_{q}-1\right\}$

*All the four possible functions are listed*.

#### 2.4.1. Multi-Level Genes

**Figure 4:**
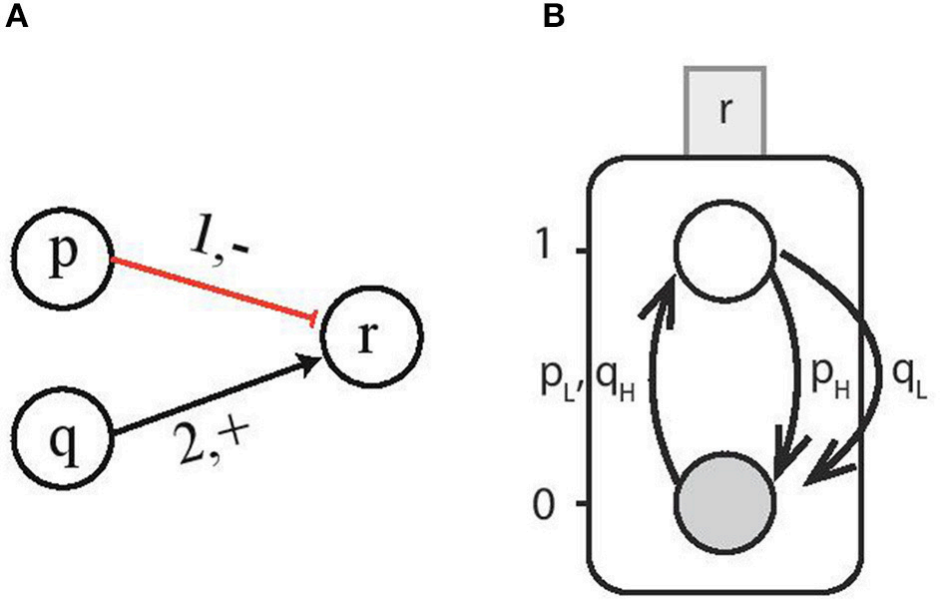
**(A)** Multi-level genes *p* and *q* act on gene *r*. For the case ¬*p* ∧ *q* and gene *p* having *l*_*p*_ = 1 and *q* having *l*_*q*_ = 2 interacting with gene *r* at threshold levels (1, −) and (2, +), respectively. **(B)** The Automata Network for gene *r* on which multi-level genes *p* and *q* are acting at the same time. The upregulation of AN for gene *r* takes place for the condition *p*_*L*_ = {*p* < 1} and *q*_*H*_ = {*q* ≥ 2}.

Now we consider the interactions involving multi-level genes which form a cooperative sort. For instance, consider gene *p* and gene *q* to have 3 and 4 threshold levels, respectively (i.e., *l*_*p*_ = 1 and *l*_*q*_ = 2), and they interact with gene *r* at threshold level *t*_*p*_ = 1 and *t*_*q*_ = 2, respectively, as shown in Figure 4A. Since the individual genes activate (*resp*. inhibit) the target gene for all the discrete levels above (*resp*. below) the threshold level, therefore, this interaction can be represented by a Boolean function with *H* containing all the discrete levels required for activation and *L* having levels for inhibition. Then for the case ¬*p* ∧ *q* which is the logical function for upregulation of gene *r*, the corresponding *Cond*_*ACT*_ is *pq*_*LH*_ which gives us the effective levels $LEV_{p}^{-}<1=\left\{0;0\right\}$ and $LEV_{q}^{+}\geq 2=\left\{2;2\right\}$. Figure 4B shows the AN for gene *r* on which multi-level genes *p* and *q* are acting at the same time. The corresponding condition for upregulation for gene *r* requires *p*_*L*_ = {*p* < 1} and *q*_*H*_ = {*q* ≥ 2}. Similarly, the downregulation takes place when either *p*_*H*_ = {*p* ≥ 1} or *q*_*L*_ = {*q* < 2}.

### 2.5. Abstraction of Switch Conditions for Gene Regulation

We intend to model the Process Hitting in a BRN with the help of concurrent finite timed automata (Alur, 1999) in which the qualitative levels of each gene would be represented by the states of its corresponding automaton and the gene evolution would be determined by the automaton dynamics. For this we need to abstract the switch conditions under which the automaton would move from one state to another.

Our approach is to directly derive the switch conditions from the effective levels corresponding to each action contained in the set H. In order to model the timed automaton representing the dynamics of gene *r*, we abstract the switch conditions which are required for it to move from one state to another. These switch conditions are abstracted directly from the Regulation conditions and effective levels.

**Definition 8. (Hit Part)**. *We abstract the hit part μ from the permissible process hits contained in* H *corresponding to up (resp. down) regulation of each gene in the following manner:*

$$\mu =\{\begin{array}{ll} p_{i}, & i\in \{L,H\},\;for\;hit\;by\;single\;gene\;\\ p_{xy}, & xy\in \{LL,LH,HL,HH\},\;for\;cooperative\;hit\; \end{array}$$

*where L* = [0; *t_p_* − 1] *and H* = [*t_p_*; *l_p_*].

Corresponding to each hit part μ, we obtain the qualitative threshold levels which permit the change in expression level of the target gene. From these levels we deduce the appropriate switch conditions ∧ which result in the dynamics of the target gene.

**Definition 9. (Switch Conditions for Gene Regulation)**. *We define Switch Conditions* ∧ *for each process hit contained in the set* H *as below:*

1. *for every hit by a single gene μ* = *p*_*i*_, *i* ∈ {*L, H*}, *the switch condition is:* $\wedge =\{\begin{array}{ll} p=\left\{0,\ldots ,t_{p}-1\right\}, & \text{ for }i=L\\ p=\left\{t_{p},\ldots ,l_{p}\right\}, & \text{ for }i=H \end{array}$
2. *for every cooperative hit μ* = *p*_*xy*_, *xy* ∈ {*LL, LH, HL, HH*}, the switch condition is: $\wedge =\{\begin{array}{l} p=\left\{0,\;...,\;t_{p}-1\right\},\;q=\left\{0,\;...,\;t_{q}-1\right\},\\ \text{ for }x=L,\;y=L\\ p=\left\{0,\;...,\;t_{p}-1\right\},\;q=\left\{t_{q},\;...,\;l_{q}\right\},\\ \text{ for }x=L,\;y=H\\ p=\left\{t_{p},\;...,\;l_{p}\right\},\;q=\left\{0,\;...,\;t_{q}-1\right\},\\ \text{ for }x=H,\;y=L\\ p=\left\{t_{p},\;...,\;l_{p}\right\},\;q=\left\{t_{q},\;...,\;l_{q}\right\},\\ \text{ for }x=H,\;y=H \end{array}$

**Example 3**. For the *transition* $r_{0}\overset{p_{0},q_{1}}{\underset{}{\to }}r_{1}$ given in *Example 3* in which the gene *r* is upregulated from *r*_0_ to *r*_1_ in the presence of *p*_0_ and *q*_1_, the effective levels are obtained as $LEV_{p}^{-}=0$ and $LEV_{q}^{+}=1$. We see that gene *r* can change its state from *r*_0_ to *r*_1_ only when gene *p* and *q* are at threshold levels 0 and 1, respectively. Therefore, the appropriate switch condition is derived as ∧ :{*p* = 0, *q* = 1}. For the multi-level genes shown in Figure 4B, the effective levels for regulation condition *Cond*_*ACT*_ of gene *r* were obtained as $LEV_{p}^{-}<1=\left\{0;0\right\}$ and $LEV_{q}^{+}\geq 2=\left\{2;3\right\}$. Thus, in this case the switch conditions would be ∧ :{(*p* = 0, *q* = 2) ∨ (*p* = 0, *q* = 3)} which are all the combinations of effective levels of genes *p* and *q* at which gene *r* is upregulated.

## 3. Hybrid Modeling

### 3.1. René Thomas' Logic Formalism

Initially, René Thomas presented a qualitative formalism based on Boolean logic applicable on Biological Regulatory Networks (Thomas, 1978, 2013) which closely approximated the ODE models. Later, the boolean model was found unable to represent various interactions taking place in BRNs at varying gene expression concentrations. This led to the presentation of kinetic logic formalism which allows the modeling of discretely abstracted concentration levels other than boolean as well (Thomas and d'Ari, 1990). It has been further enriched with Parametric Time Delays in Hybrid Modeling (Ahmad et al., 2006).

### 3.2. Hybrid Modeling Framework

**Figure 5:**
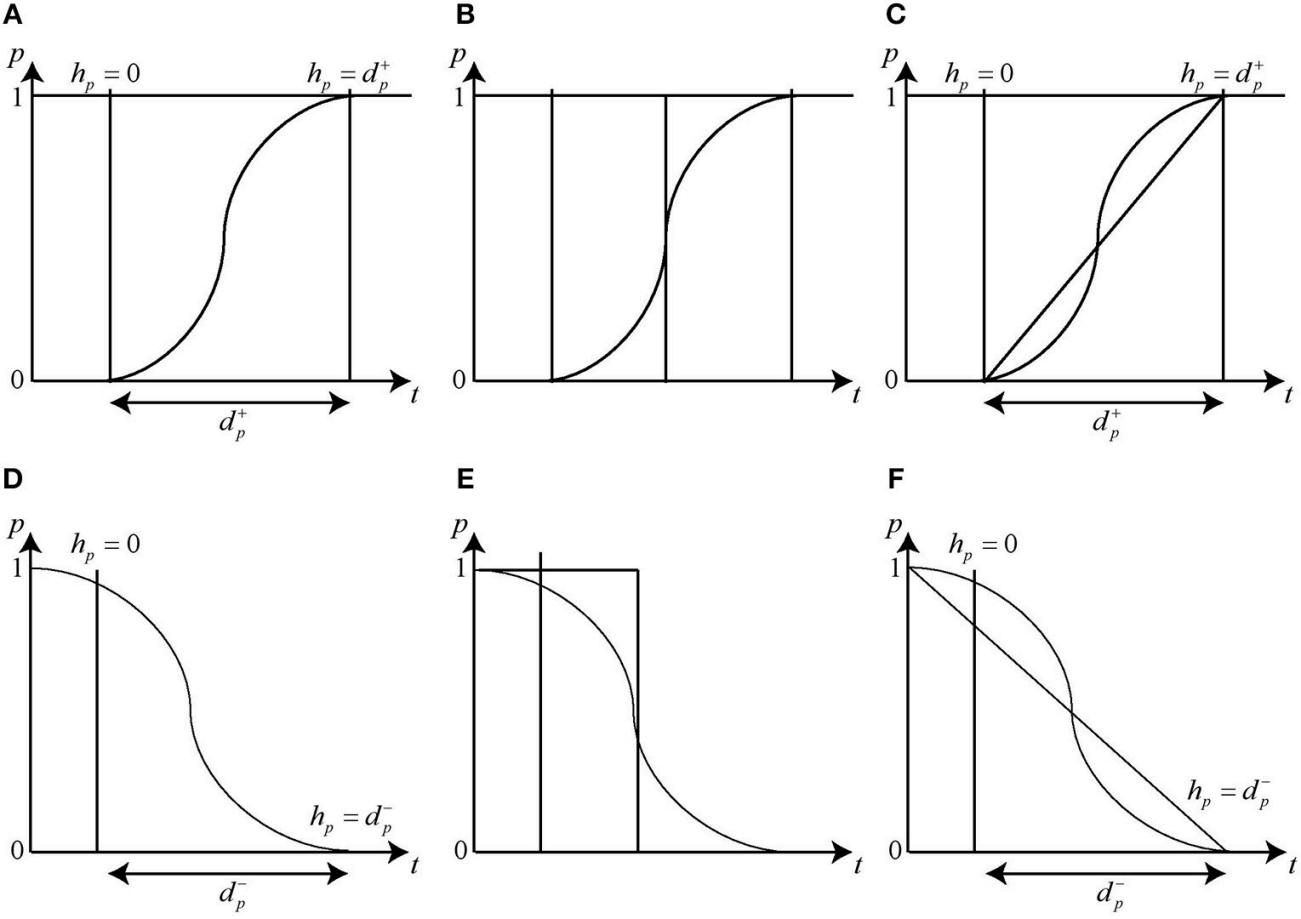
Activation and Inhibition delays. The clock variable *h*_*p*_ measures the time of evolution between two discrete levels. Initially the clock is set to zero and the changes in the level occurs in a delay time $d_{p}^{+/-}$. The change in expression level during Gene Activation is represented by **(A)** Sigmoidal function **(B)** Step function and **(C)** Piece-wise Linear function. Similarly, the change in expression level during Gene Inhibition is represented by **(D)** Sigmoidal function **(E)** Step function and **(F)** Piece-wise Linear function.

It is difficult to model the changes in concentration levels of a protein through the discrete modeling frameworks. For instance, the protein expression level which often follows a sigmoidal curve (Figure 5A) is represented by positive integers in a discrete model where the change in level from 0 to 1 takes place instantly (Figure 5B) which is not a true representation of the actual increase in concentration of protein in a cellular environment. Therefore, the changes in concentration expression levels were proposed to be represented by piecewise linear curves for the modeling of the sigmoidal nature of protein expression (Ahmad et al., 2008) as shown in Figure 5C, as opposed to the step functions used in discrete models. Figures 5D–F) depict the modeling in case of degradation of protein.

A clock variable *h*_*p*_ is associated with each protein to measure the time it takes (delay) to go from a particular expression level to the next one. Initially, the value of each delay *h*_*p*_ is set to zero, which then increase to $d_{p}^{+}$ (resp. $d_{p}^{-}$) that signifies the *production* (resp. *degradation*) delay, i.e., the time it takes to *increase* (resp. *decrease*) the concentration level of the particular protein by 1 threshold level. Evolution rate for each clock *h*_*p*_ is set using first order derivative *dh*_*p*_/*dt* = *x* where *x* ∈ {0, 1, −1} which represents no change, increase or decrease, respectively (Ahmad et al., 2008). Since the exact value of the delays associated with the production or degradation of proteins is not known in most cases, therefore, unvalued parametric delays are used instead and are determined through hybrid modeling (Ahmad et al., 2012). Hybrid modeling approach has been applied for behavioral modeling of several biological networks including MAL-associated network (Ahmad et al., 2012), dengue virus pathogenesis and clearance mechanism (Aslam et al., 2014), tail-resorption in tadpole's metamorphosis (Khalis et al., 2009), role of O-linked N-acetylglucosamine transferase in oncogenesis and cancer progression in hexosamine biosynthetic pathway (Saeed et al., 2016) and immunity control mechanism in bacteriophage lambda (Richard et al., 2006).

The hybrid modeling method has also been applied in opinion evolution in social networks (Shang, 2018). The Hybrid consensus for averager-copier voter networks with non-rational agents has been modeled and it is shown that there is a distinct influence of voters on the consensus and evolution of the opinion process.

The delays are of two types; Signal Transmission Delay and Signal Processing Delay. While the outcome for both types of delay would be the same, however, the processing delay is likely to cause errors in the consensus behavior whereas it is independent of the transmission delay (Shang, 2017). For study of BRNs we have to deal with the processing delay, hence, keeping track of the transient changes during the delay period is important and essentially required to be taken into account for correctly modeling the evolution of BRN and understanding its dynamical behavior.

### 3.3. Parametric Bio Linear Hybrid Automaton

We formally define hybrid automaton here by mainly using the notations and definitions given in Alur (1999) and adapted from Ahmad et al. (2006). We use a set of *clock* variables *X* = {*h*_1_, …, *h*_*n*_} to represent time which progress synchronously in accordance with $\dot{h_{i}}=1$ or −1 and can be reset to zero. The clock constraints *φ* for the set *ϕ*(*X*) is defined by the grammar:

$$\varphi ::=\text{true}|h\leq d|h\geq d|h=d|\varphi_{1}\wedge \varphi_{2}$$

where *h* ∈ *X* and *d* is an un-valued parameter.

**Definition 10. (Parametric Biological Linear Hybrid Automaton (Bio-LHA))**. *A parametric Bio linear hybrid automaton* 𝔹 *is a tuple* (*L, l*_0_, *D, X, E, Inv, Dif*) *where*,

- *L* *is a finite set of locations*,
- *l*_0_ ∈ *L* *is the initial location*,
- *D* *is a finite set of parameters (delays)*,
- *X* *is a finite set of real-valued variable (clocks)*,
- *E* ⊆ *L* × φ × 2^*X*^ × *L* *is a finite set of edges with typical element* *e* = (*l, g, R, l*′) ∈ *E* *representing an edge from* *l* *to* *l*′ *with guard* *g* *(clock constraint of the form* *h* = *d) and the reset set* *R* ⊆ *X*,
- *Inv*: *L* → φ *assigns an invariant to any location*,
- *Dif*: *L* × *X* → {−1, 0, 1} *maps each pair* (*l, h*) *to an evolution rate*.

The Transition System related semantics of the parametric Bio-LHA is given below according to the time domain 𝕋, where 𝕋^*^ = 𝕋\{0}.

**Definition 11. (Semantics of Bio-LHA)**. *Let* γ *be a valuation for the parameters* *P* *and* ν *represents the values of clocks in a location. The* (𝕋, γ)*-semantics of a parametric Bio-LHA* 𝔹 = (*L, l*_0_, *P, X, E, inv, dif*) *is defined as a timed transition system*_*B*_ = (𝕊, *s*_0_, 𝕋, → ) *where:*

1. 𝕊 = {(*l*, ν)|*l* ∈ *L* *and* ν ⊧ *Inv*(*l*)};
2. *s*_0_ *is the initial state and*
3. *the relation* → ⊆ 𝕊 × 𝕋 × 𝕊 *is defined for* *t* ∈ 𝕋 *as:*

- *discrete transitions:* $\left(\ell ,\nu \right)\overset{\;0\;}{\underset{}{\to }}\left(\ell^{\prime },\nu^{\prime }\right)$ iff ∃(ℓ, *g, R*, ℓ′) ∈ *E* *such that* *g*(ν) = *true*, ν′(*h*) = 0 if *h* ∈ *R* *and* ν′(*h*) = ν(*h*) *if* *h* ∉ *R*.
- *continuous transitions: For* *t* ∈ T^*^, $\left(\ell ,\nu \right)\overset{\;t\;}{\underset{}{\to }}\left(\ell^{\prime },\nu^{\prime }\right)$ *iff* ℓ′ = ℓ, ν′(*h*) = ν(*h*) + *Dif*(ℓ, *h*) × *t*, *and for every* *t*′ ∈ [0, *t*], (ν(*h*) + *Dif*(ℓ, *h*) × *t*′⊧*Inv*(*l*), *where* ⊧ *represents satisfaction operator*.

### 3.4. Network of Bio-LHA

A BRN is composed of various entities which evolve concurrently depending on the activating (*resp. inhibiting*) influence of other entities as defined in the BRN. We have represented each entity with a Bio-LHA and its dynamics is also defined above. Next we define the network of Hybrid Automaton 𝔹_*N*_ which is built up from composition of individual Bio-LHA 𝔹_1_, …, 𝔹_*n*_.

**Definition 12. (Network of Bio-LHA)**. *Let* 𝔹_*i*_: = (*L*_*i*_, *l*_0, *i*_, *D*_*i*_, *X*_*i*_, *E*_*i*_, *Inv*_*i*_, *Dif*_*i*_), *i* ∈ 1, …, *n* *be the component Bio-LHA representing the genes of the BRN. The network of Bio-LHA representing* *n* *genes is* 𝔸 = (*L, l*_0_, *D, X, E, Inv, Dif*) *where*,

- *L* = *L*_1_×… × *L*_*n*_, *is the set of locations*,
- *l*_0_ = (*l*_0, 1_…, *l*_0, *n*_) ∈ *L* *is the initial location*,
- $D=\overset{n}{\underset{i=1}{\cup }}D_{i}$, *is the set of delays*,
- $X=\overset{n}{\underset{i=1}{\cup }}X_{i}$, *is the set of clocks*,
- The transition relation E is defined by the following asynchronous rule:
     *If l*_*i*_ *is any component location of l* ∈ *L then* $\left(\left(\ldots l_{i},\ldots \right),g_{i},R_{i},\left(\ldots l_{i}^{\prime },\ldots \right)\right)$ ∈ *E for* $\left(l_{i},g_{i},R_{i},l_{i}^{\prime }\right)\in E_{i}$ *and all other component locations of l do not change. The guard g*_*i*_ *is the conjuction of switch condition* (∧) *and clock constraint* (φ),
- *Inv* : $L\to \overset{n}{\underset{i=1}{\cup }}$, *Inv_i_*(*l*), *is mapping of invariants to locations*,
- *Dif* : *L*×*X* → {−1, 0, 1}, *is the mapping of clocks to evolution rates*.

### 3.5. Representation of Hybrid Automaton

**Figure 6:**
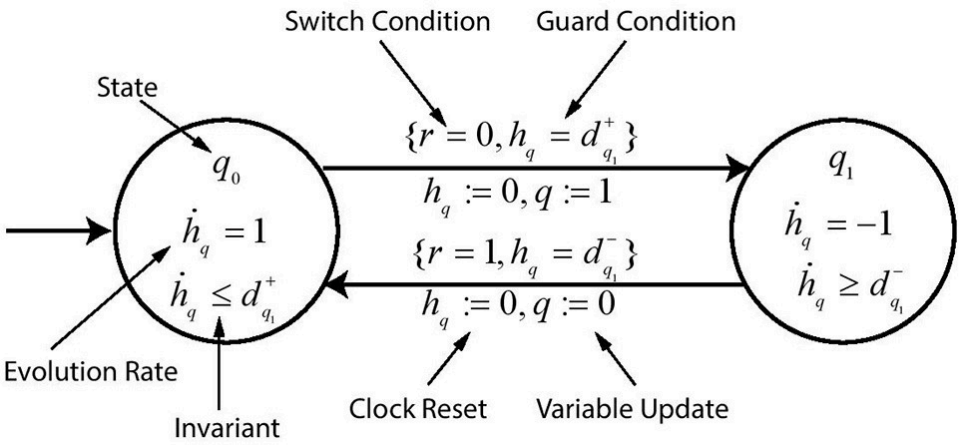
A toy example of hybrid automaton. Various labels describe the function of each symbol used in the automaton.

The modeling of Hybrid Automaton representing evolution of gene *q* is depicted in Figure 6 which is inhibited by gene *r*. The set of transitions H is given by $\left\{q_{0}\overset{r_{0}}{\underset{}{\to }}q_{1}\right\},\left\{q_{1}\overset{r_{1}}{\underset{}{\to }}q_{0}\right\}$. The automaton is modeled with two states *q*_0_ and *q*_1_ which represents the discrete threshold levels of the gene *q*. Each state is labeled with its evolution rate $d_{q_{1}}^{+/-}$ and its *invariant* for which the automaton remains in that state. The edges are labeled with the *guard* condition on the clock variable as well as the switch condition ∧ on the gene level $LEV_{q}^{+/-}$. When the *guard* becomes *True*, the automaton moves to the next state with the clock being reset and also updating the variable *q* which contains the current value of gene threshold level.

### 3.6. Implementation of Hybrid Automaton With HyTech

HyTech (Henzinger et al., 1997) is a model checking tool developed for the formal verification of real-time systems. It is suitable for modeling of Linear Hybrid Automata and has a powerful set of analysis commands as well as parametric synthesis capability. Using HyTech for modeling of BRN, each gene is modeled by an automaton. Thus, the current state of the BRN would be given by the current state of all the automata considered together.

**Example 4**. The code for implementation of toy example of hybrid automaton *geneQ* shown in Figure 6 in HyTech is given below. Keyword *loc* is used to specify the *state* of automaton (e.g., *q*_0_), keyword *while* is followed by the *invariant* condition (e.g., *hq* <= *dpq*1), keyword *wait* is used to give the rate of increase or decrease during the transition (e.g., {*dhq* = 1}), keyword *when* is followed by the *guard* condition (e.g., *r* = 0 & *hq* = *dpq*1), keyword *do* is used for updating of the state variable (e.g., {*hq*′ = 0, *q*′ = 1}) and keyword *goto* is followed by the next state of the automaton (e.g., *q*_1_).


automaton geneQ
synclabs: ;
initially q0 & q=0 & hq=0;
loc q0: while hq < =dpq1 wait {dhq=1}
when r=0 & hq=dpq1 do {hq'=0,q'=1} goto q1;
loc q1: while hq < =dnq1 wait {dhq=-1}
when r=1 & hq=dnq1 do {hq'=0,q'=0} goto q0;
end --geneQ


We use the parametric delay variable *hq* here with all the transitions between different states of automaton which evolve at some rate till the switch condition ∧ becomes true for respective transitions. In this way we are able to get the range of values for this delay variable for the automaton to move from one state to another. Accordingly, we are able to determine the constraints on the delay variable corresponding to each state of automaton. When the system of automata representing the complete interaction graph of BRN is composed with switch conditions and delay variables for each gene evolution, we get the constraints on delay variables for each of the composed state of automata. The complete methodology is given in next section.

## 4. Incorporating Time Delays in Process Hitting Framework

The aim of this section is to give the methodology of our proposed *Hybrid Process Hitting* technique in which we have incorporated the Time Delays from Hybrid Modeling described above into the Process Hitting Framework.

### 4.1. Obtaining Process Hits for BRN

**Figure 7:**
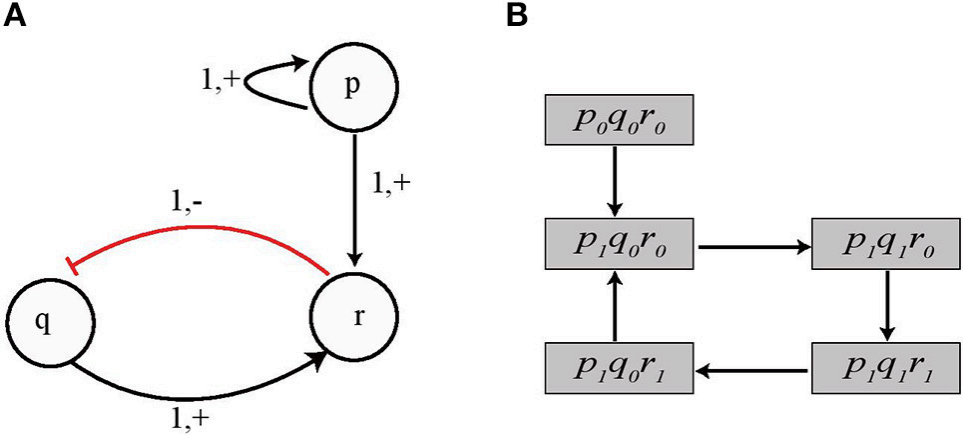
**(A)** An example BRN in which three genes *p, q*, and *r* interact with each other. Gene *p* and *q* are the activators of gene *r* whereas gene *r* inhibits gene *q*. **(B)** State Transition Graph for the toy BRN is shown in which the system of automata comprising of genes *p, q*, and *r* completes a cycle from state 〈1, 0, 0〉 through states 〈1, 1, 0〉, 〈1, 1, 1〉, and 〈1, 0, 1〉 .

Starting with the Interaction Graph of a BRN which specifies all the regulations *(type and threshold level)* of all its genes on each other, all the local transitions in the BRN were captured through application of Process Hitting as a first step. We now consider an example BRN in which two individual genes *p* and *q* are interacting with gene *r* as shown in Figure 7A. By considering the permissible actions in the generalized dynamics in this BRN we get the possible local transitions H as given below:

$$H=\left\{q_{0}\overset{\left\{r_{0}\right\}}{\to }q_{1},\;q_{1}\overset{\left\{r_{1}\right\}}{\to }q_{0},\;r_{0}\overset{\left\{p_{1},q_{1}\right\}}{\to }r_{1},\;r_{1}\overset{\left\{p_{0}\right\},\left\{q_{0}\right\}}{\to }r_{0}\right\}.$$

### 4.2. Deducing the Switch Conditions and Effective Levels

Now using Table 1, the effective levels of interacting genes *p* and *q* are determined from the corresponding condition required by the cooperative sort for upregulation of gene *r*. For *pq*_11_ we get $LEV_{p}^{+}=\left\{1\right\}$ for gene *p* and $LEV_{q}^{+}=\left\{1\right\}$ for gene *q*. We interpret the values of effective levels in a straight forward manner in which the levels represent the current state of the individual automaton of gene *p* and *q*. In this case, the automaton *geneP* is at state *P*_1_ and automaton *geneQ* is at state *Q*_1_. For automaton *geneR* which describes the dynamics of gene *r*, the effective levels, therefore, specifies the *guard* condition in terms of exact combination of state variables of other automata that would induce the transition from state *R*_0_ to *R*_1_.

### 4.3. State Transition Graph

All the automata when considered together would represent the complete model of BRN. Hence, the transitions from one state to another would be governed by the dynamics of the modeled BRN. At any given time, the locations of all the automata give us the current state of the BRN. A change in location of any one automata result in change of state of BRN. For the BRN shown in Figure 7A consisting of genes *p, q* and *r* with expression levels as 1, 0 and ), respectively, the current state is denoted as 〈1, 0, 0〉 with corresponding locations of automata as *loc* *p*_1_, *loc* *q*_0_ and *loc* *r*_0_. Here, we note that the switch condition for evolution of automaton *geneQ* is satisfied (i.e., $LEV_{r}^{+}=\left\{0\right\}$) for it to move from location *loc* *q*_0_ to *loc* *q*_1_, thus the next state of the system would be 〈1, 1, 0〉.

In the state 〈1, 0, 0〉, it may be noted that the required switch conditions for evolution of automaton *geneR* were not satisfied, so this automaton could not evolve. However, we see that in state 〈1, 1, 0〉 the switch conditions for evolution of *geneR* are now satisfied (i.e., *p* = 1 and *q* = 1) therefore, the next transition lead the system to state 〈1, 1, 1〉. Subsequently, the system evolves to state 〈1, 0, 1〉 from where it comes back to state 〈1, 0, 0〉. The corresponding state transition graph for the dynamics of the system model is shown in Figure 7B.

### 4.4. Enriching the Automaton With Time Delays

The transition from one state of the system to another takes place when the concentration level increases (*resp. decreases*) from one threshold level to another and it takes a finite amount of time. This time is measured in terms of delay variable $d_{q}^{+/-}$ in hybrid modeling of BRN. We note that the automaton moves from one state to another instantaneously once the switch conditions are satisfied. So in order to measure the time delay for this transition we introduce the *parametric* clock variable *hq* which increases at a rate *dhq*/*dt* for the time the automaton remains in a particular state and its value is recorded on transition to the next state.

Consider the initial location of automaton *geneQ* is *loc* *q*_0_ and the clock variable *h*_*q*_ is initially set to *0* which starts to increase with rate *dhq*/*dt*. On satisfaction of switch conditions, automaton *geneQ* leaves its current state *loc* *q*_0_ and moves to next state *loc* *q*_1_ which represents the transition of Gene *q* from expression level 0 to 1. At this time the value of clock variable *h*_*q*_ for this transition is noted in terms of delay variable as $d_{q_{1}}^{+}$. Similarly, the delay for moving from level 1 to 0 will be given by $d_{q_{1}}^{-}$.

### 4.5. Composing System of Automata

We compose the System of Automata by considering all individual automaton together. The transitions in each automaton are linked with the state of other automata through switch conditions and the time taken by each transition is obtained in terms of its delay variable. We recall that the switch conditions are given by the *refined cooperative hits* and represent the permissible dynamics in the BRN. When all the individual automaton are considered together we obtain the constraints on the range of values of respective clocks for the system to move from one state to another.

**Figure 8:**
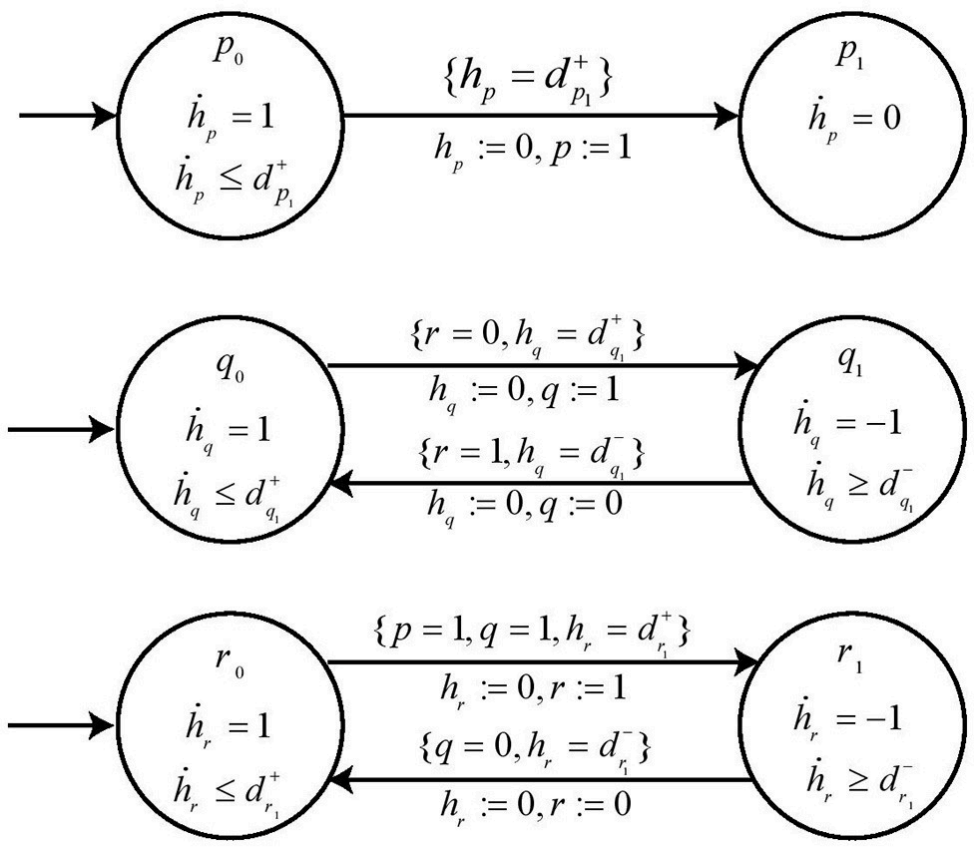
The individual automaton modeling the behavior of genes *p, q*, and *r*. Each automata contains the respective *guard* and *switch* conditions.

**Example 5**. In order to describe our methodology for obtaining the time delays, we now model the individual genes in BRN of Figure 7A with respective automaton. From H we note the *action* (switch condition) for upregulation *q*_0_ to *q*_1_ for automaton *geneQ* as *r*_0_ and effective level $LEV_{r}^{+}=\left\{0\right\}$. Therefore, the *guard* condition for automaton *geneQ* is set as *r* = 0 & $h_{q}=d_{q_{1}}^{+}$. Similarly for downregulation, the *guard* condition is *r* = 1 & $h_{q}=d_{q_{1}}^{-}$. In case of *geneR*, we have the switch condition for upregulation as *pq*_11_ given by the logical function *p* ∧ *q* with effective levels as $LEV_{p}^{+}=\left\{1\right\}$ and $LEV_{q}^{+}=\left\{1\right\}$. For downregulation, we get the switch conditions and effective levels from Table 1 for the given logical function. Accordingly, the *guard* conditions in automaton *geneR* are set to model this behavior. The individual automaton for *p, q* and *r* are modeled as shown in Figure 8. The corresponding *hytech* file for modeling this BRN is given in Supplementary File 1. We assume that *gene p* is at level 1 initially and *hytech* file is run to get the constraints on delay parameters corresponding to each state of composed system as shown in Table 2 below.

**Table 2:** Delay Constraints obtained for the example BRN through modeling of its corresponding system of Automata in HyTech.

**Path**	***p*_0_*q*_0_*r*_0_→*p*_1_*q*_0_*r*_0_→*p*_1_*q*_1_*r*_0_→*p*_1_*q*_1_*r*_1_→*p*_1_*q*_0_*r*_1_→*p*_1_*q*_0_*r*_0_**
**Delay**	$\left(d_{p_{1}}^{+}\leq d_{q_{1}}^{+}\wedge d_{p_{1}}^{+}\leq d_{r_{1}}^{+}\right)\wedge \left(d_{q_{1}}^{+}\leq d_{p_{1}}^{+}\wedge d_{p_{1}}^{+}\leq d_{q_{1}}^{+}+|d_{q_{1}}^{-}|\right)\;\wedge$
**Constraints**	$\left(d_{r_{1}}^{+}\leq d_{q_{1}}^{+}+|d_{q_{1}}^{-}|\right)\wedge \left(d_{q_{1}}^{+}+|d_{q_{1}}^{-}|\leq d_{r_{1}}^{+}+|d_{r_{1}}^{-}|\right)\;\wedge$
	$\left(d_{r_{1}}^{+}+|d_{r_{1}}^{-}|\leq 2d_{q_{1}}^{+}+|d_{q_{1}}^{-}|\right)$

*The parametric delays have been introduced in Process Hitting Framework to give the constraints for the complete path of the State Transition Graph*.

### 4.6. Inference of Parametric Delays in *Hybrid Process Hitting*

The range of delay parameters alongwith the constraints on delay variables in terms of time delays is obtained as output from simulation of System of Automata which modeled the proposed *Hybrid Process Hitting* approach. As evident from Table 2, for each path or trajectory from one particular state to another of the State Transition Graph, we get the constraints on clock variables in terms of time delays for each gene of BRN. For instance, the delay constraint for the path from state *p*_0_*q*_0_*r*_0_ to state *p*_1_*q*_0_*r*_0_ requires that $d_{p_{1}}^{+}\leq d_{q_{1}}^{+}$ and $d_{p_{1}}^{+}\leq d_{r_{1}}^{+}$. Similarly, the delay constraints corresponding to the paths between various states are obtained from running the *HyTech* model.

It is highlighted that the delays $d_{q_{1}}^{+}$ and $d_{q_{1}}^{-}$ represents the accumulation and degradation time of gene *q*. Hence, the constraints on the delays give us the conditions in terms of time taken for accumulation or degradation of biological entities in the BRN and clearly define the dynamics of the modeled system.

We are thus able to determine the dynamical behavior of a biological regulatory network in which the constraints on time delays are deduced for each state.

## 5. Biological Application I: Bacteriophage Lambda

In this section we apply the proposed *Hybrid Process Hitting* approach to the well-known example of *Bacteriophage* λ (Thieffry and Thomas, 1995). This network has been widely studied because of its peculiar switch mechanism because of which this virus chooses between *lysis* and *lysogenization*. These two responses are the result of different dynamics of the BRN and thus leads to separate attractors.

### 5.1. Automata Network

**Figure 9:**
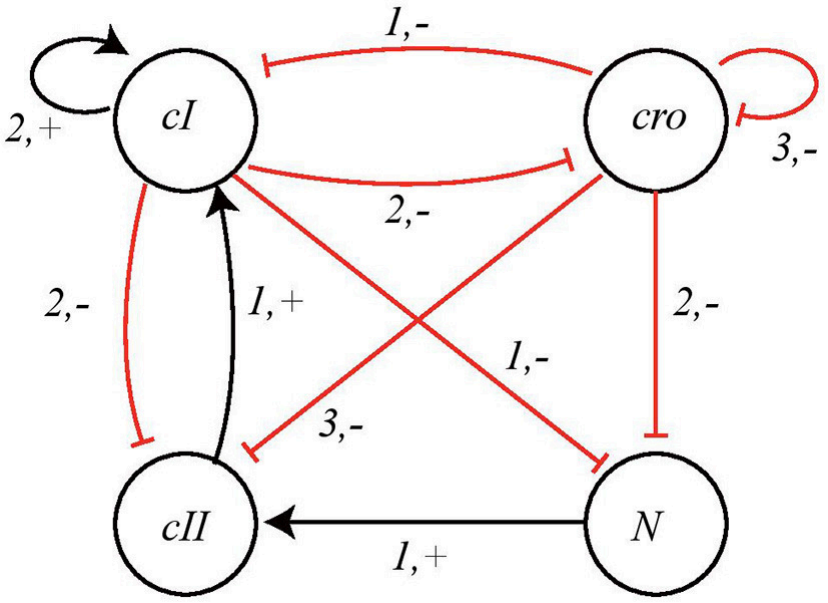
BRN of Bacteriophage λ immunity response showing interactions between nodes cI, cro, cII, and N.

We take into consideration the BRN model proposed in Thieffry and Thomas (1995) and shown in Figure 9. The network has 4 nodes, having expression levels 3, 4, 2, and 2, respectively, so in this case:

Σ = {*cI, cro, cII, N*}, *L*_*cI*_ = {*cI*_0_, *cI*_1_, *cI*_2_},
  *L*_*cro*_ = {*cro*_0_, *cro*_1_, *cro*_2_, *cro*_3_}, *L*_*cII*_ = {*cII*_0_, *cII*_1_} and
  *L*_*N*_ = {*N*_0_, *N*_1_}.

**Figure 10:**
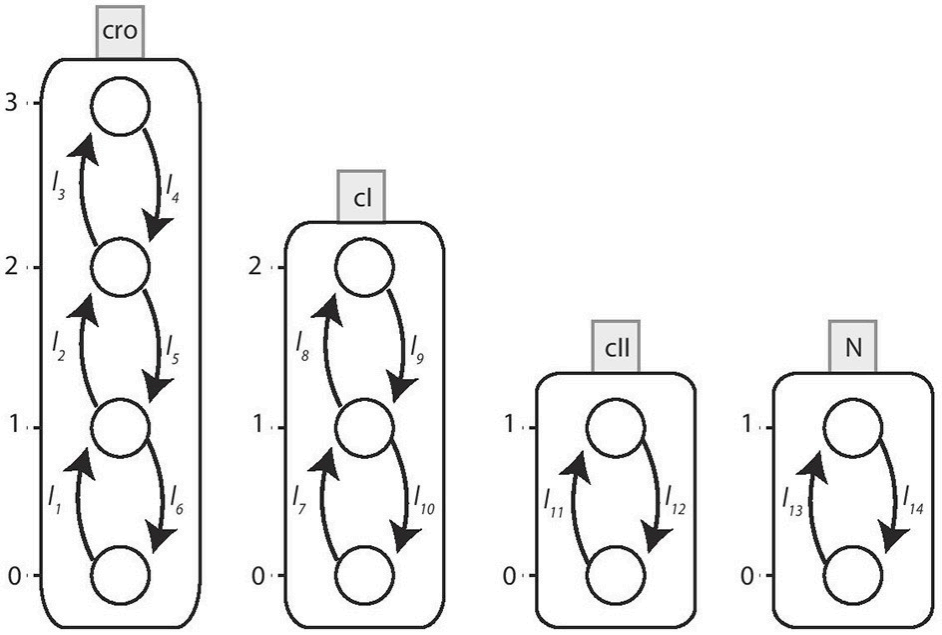
The Automata Network model of Bacteriophage λ immunity response. The corresponding local transitions H are: *l*_1_ = *l*_2_ = *l*_3_ = {*cI*_≤ 1_}, *l*_4_ = {*cI*_2_, *cro*_3_}, *l*_5_ = *l*_6_ = {*cI*_2_}*l*_7_ = *l*_8_ = {*cII*_1_, *cro*_0_}, *l*_9_ = *l*_10_ = {*cII*_0_}, {*cro*_ ≥ 1_}*l*_11_ = {*N*_1_, *cI*_≤ 1_, *cro*_≤ 2_}, *l*_12_ = {*N*_0_}, {*cI*_2_}, {*cro*_3_}*l*_13_ = {*cI*_ < 1_, *cro*_ < 2_}, *l*_14_ = {*cI*_ ≥ 1_}, {*cro*_ ≥ 2_}.

We determine the *local transitions* H for this network from the BRN interaction graph. The associated Automata Network is shown in Figure 10.

### 5.2. Obtaining the Switch Conditions

The logical function for the cooperation between *cI* and *cro* is ¬*cI* ∧ ¬*cro* as both nodes are inhibitors of node *N* which implies that the process *cI*−*cro*_*LL*_ produce upregulation of node *N* while all other processes, i.e., *cI*−*cro*_*LH*_, *cI*−*cro*_*HL*_, and *cI*−*cro*_*HH*_ would result in downregulation of target node *N*. Here we note that the nodes *cI* and *cro* are having multiple expression levels but the threshold level for interactions on node *N* are 1 and 2, respectively. This condition then gives us the *effective levels* for the process *cI*−*cro*_*LL*_ which are $LEV_{cI}^{-}=\left\{0\right\}$ and $LEV_{cro}^{-}=\left\{0,1\right\}$. Thus, the gene *N* would bounce from *N*_0_ to *N*_1_ if its regulators have the expression levels (*cI*_0_, *cro*_0_) or (*cI*_0_, *cro*_1_).

Similarly, we get the effective levels and corresponding expression levels for downregulation of node *N* from the other processes of cooperative sort *cI*−*cro* as follows:

$cI-cro_{LH}:LEV_{cI}^{-}=\left\{0\right\},LEV_{cro}^{+}=\left\{2,3\right\}$ (*cI*_0_, *cro*_2_), (*cI*_0_, *cro*_3_)

$cI-cro_{HL}:LEV_{cI}^{+}=\left\{1,2\right\},LEV_{cro}^{-}=\left\{0,1\right\}\left(cI_{1},cro_{0}\right),\left(cI_{1},cro_{1}\right),\left(cI_{2},cro_{0}\right),\left(cI_{2},cro_{1}\right)$

$cI-cro_{HH}:LEV_{cI}^{+}=\left\{1,2\right\},LEV_{cro}^{+}=\left\{2,3\right\}\left(cI_{1},cro_{2}\right),\left(cI_{1},cro_{3}\right),\left(cI_{2},cro_{2}\right),\left(cI_{2},cro_{3}\right)$

### 5.3. Automaton Specification

**Figure 11:**
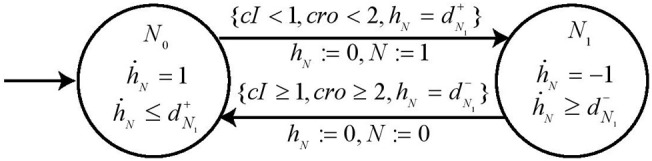
Automaton for node *N*. The automaton is constructed to represent the dynamics in node N due to its activators (resp. inhibitors) which cooperate under the logical function ¬*cI* ∧ ¬*cro* and corresponding switch conditions. The automaton is also enriched with parametric clock variable *dN* which gives us the measure of time taken for the automaton to change states in terms of delays $d_{N_{1}}^{+}$ (*resp*. $d_{N_{1}}^{-}$).

These expression levels give us the required switch conditions for completely specifying the automaton for node *N*. It contains two states, namely, *loc* *N*_0_ and *loc* *N*_1_ since node *N* has two processes and are represented by state variable *N* = 0 and *N* = 1. The clock variable is *h*_*N*_ and the parametric delay variables are $d_{N_{1}}^{+}$ (*resp*. $d_{N_{1}}^{-}$) for the time taken for up (*resp*. down) regulation for node *N*. By following our proposed methodology we deduce the *Invariant* and *guard* conditions for each state of the automaton to get the *Hybrid Process Hitting* model for node *N* as shown in Figure 11.

**Figure 12:**
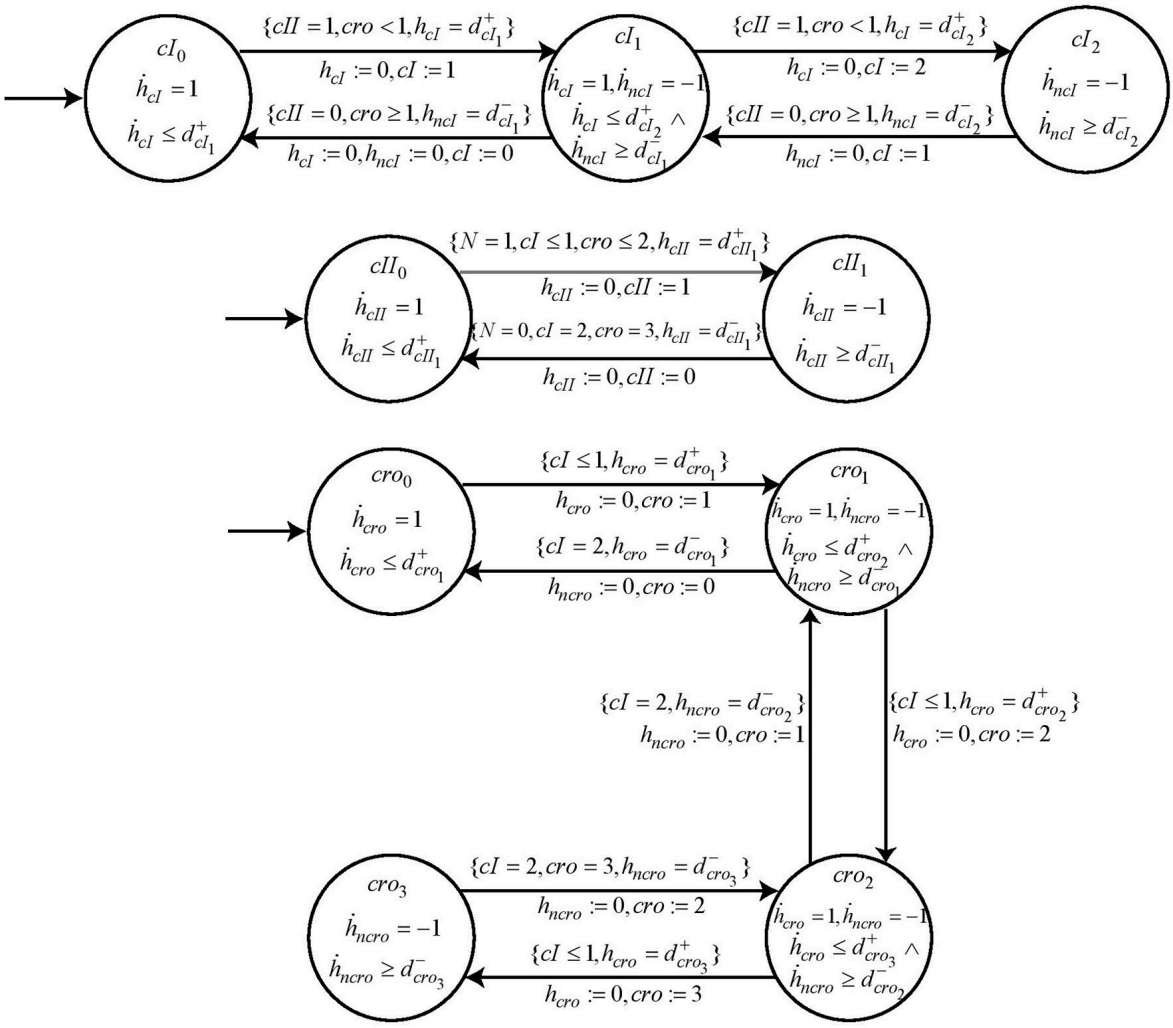
The individual automaton modeling the behavior of genes *cI, cII*, and *cro*. Each automata contains the respective *guard* and *switch* conditions.

Following the same methodology, the automaton for other nodes *cI*, *cro* and *cII* are constructed with their switch conditions obtained from the logical functions ¬*cI* ∧ ¬*cro* ∧ *cII*, ¬*cI* ∧ ¬*cro* and ¬*cI* ∧ ¬*cro* ∧ *N*, respectively. These logical functions are the result of refined cooperative hits obtained from Process Hitting. The cooperativity among three nodes as required for nodes *cI* and *cII* is constructed in two steps as explained earlier in section 2 above. The specifications of these automata are completed by introducing the respective parametric clock and delay variables. The automata for these other entities, *cI, cII* and *cro* are shown in Figure 12.

### 5.4. Modeling of Clock Variables for Multi-Level Genes

In the Bacteriophage λ BRN we have two multi-level genes, i.e., *cI* and *cro* which have 3 and 4 levels, respectively. For modeling these multi-level genes in *HyTech*, we introduce new clock variable *h*_*n*_. Consider the Automaton of gene *cI* which starts from initial location *cI*_0_ and moves to next location *cI*_1_ when the appropriate switch conditions (*cII* = 1, *cro* < 1) are met. Here if the switch conditions remain the same, the automaton would move to location *cI*_2_, however if either of the switch condition changes then the automaton would move towards location *cI*_0_. We note that the jump from *cI*_1_ to *cI*_2_ represents the accumulation of *cI* whereas moving from *cI*_1_ to *cI*_0_ shows the degradation of entity *cI*. Hence, we require two clock variables *h*_*cI*_ and *h*_*ncI*_ to keep track of the accumulation and degradation delays, respectively.

### 5.5. Composition of System of Automata

Once all the individual automaton are constructed, we now compose the system of automata by executing the *HyTech* file. Since we are interested in the dynamical characteristics of the transitions that take place, therefore, we use the *reach* command in the *Analysis* section of *HyTech*. So starting from any arbitrary initial state we are able to find the next states that the system of automata can reach alongwith the corresponding constraints on the time taken for this change of state in terms of *time delays*.

### 5.6. Results

We compose the system of automata for *Bacteriophage* λ and get the results on the constraints of time delays corresponding to each state that the system can reach as given in Supplementary File 2. It is highlighted that we get 42 states instead of the total 48 possible states which shows that the state transition graph has been reduced through application of *Process Hitting*. Since we used the *reach forward* command, therefore, the *HyTech* has computed all trajectories alongwith the delay constraints for the system of automata which it can possibly traverse with the defined *switch conditions* for each automaton.

We know that the *phage* λ BRN has two attractors, i.e., *lysogenic* corresponding to state 〈*cI, cro, cII, N*〉 = 〈2, 0, 0, 0〉 and *lysis* alternating between states 〈*cI, cro, cII, N*〉 = 〈0, 2, 0, 0〉 and 〈0, 3, 0, 0〉 (Thieffry and Thomas, 1995). From the results, we note the constraints on time delays for the paths leading to above mentioned states. For *lysogenic* condition 〈2, 0, 0, 0〉, gene *cI* reaches its highest threshold level of 2 and represses the expression of all other genes. The time delays for the path leading to this state are found to be:

$d_{cI_{2}}^{+}\leq |d_{cI_{1}}^{-}|\wedge d_{cI_{1}}^{+}+d_{cI_{2}}^{+}\leq d_{cro_{1}}^{+}\wedge d_{cI_{2}}^{+}\leq d_{cII_{1}}^{+}\wedge d_{cI_{1}}^{+}\leq d_{cII_{1}}^{+}$

In *lysis*, the delays for the path leading to state 〈0, 2, 0, 0〉 are:

$d_{cro_{2}}^{+}\leq |d_{cro_{1}}^{-}|\wedge d_{cro_{2}}^{+}\leq d_{cII_{1}}^{+}\wedge d_{cro_{1}}^{+}\leq d_{cII_{1}}^{+}\wedge d_{cro_{1}}^{+}+d_{cro_{2}}^{+}\leq d_{N_{1}}^{+}.$

and for the path leading to state 〈0, 3, 0, 0〉 the delays are:

$d_{cro_{2}}^{+}\leq |d_{cro_{1}}^{-}|\wedge d_{cro_{3}}^{+}\leq |d_{cro_{2}}^{-}|\wedge d_{cro_{2}}^{+}\leq d_{cII_{1}}^{+}\wedge d_{cro_{1}}^{+}\leq d_{cII_{1}}^{+}\wedge$

$d_{cro_{1}}^{+}+d_{cro_{2}}^{+}+d_{cro_{3}}^{+}\leq d_{N_{1}}^{+}\wedge d_{cro_{3}}^{+}\leq d_{cII_{1}}^{+}\wedge$

$2d_{cro_{1}}^{+}+2d_{cro_{2}}^{+}+2d_{cro_{3}}^{+}\leq d_{cI_{1}}^{+}+2d_{cII_{1}}^{+}\wedge d_{cro_{1}}^{+}+d_{cro_{2}}^{+}+d_{cro_{3}}^{+}\leq 2d_{cII_{1}}^{+}$.

By analysing the delay constraints for each state we get the relationship between the rates of accumulation or decay of a particular entity in the BRN. These results on delay constraints are found to be slightly different from Ahmad et al. (2008) due to the difference in the way the delays are modeled. In Ahmad et al. (2008), the time delays between various levels of af a multi-level gene were modeled with the same delay variable whereas we have specified it to be different for each change in level. For instance, our modeling methodology considers $d_{cro_{1}}^{+}$ as the time delay for *cro* to change from level *0* to *1* while $d_{cro_{2}}^{+}$ gives the time delay from level *1* to *2*. But in Ahmad et al. (2008), both these delays were modeled with the delay variable $d_{cro}^{+}$. Our specification of time delays is considered more suitable for modeling of dynamic behavior of genes in BRNs since the time for change amongst various levels of a biological entity are usually different from each other.

## 6. Biological Application II: ERBB Receptor-Regulated G1/S Transition BRN

**Figure 13:**
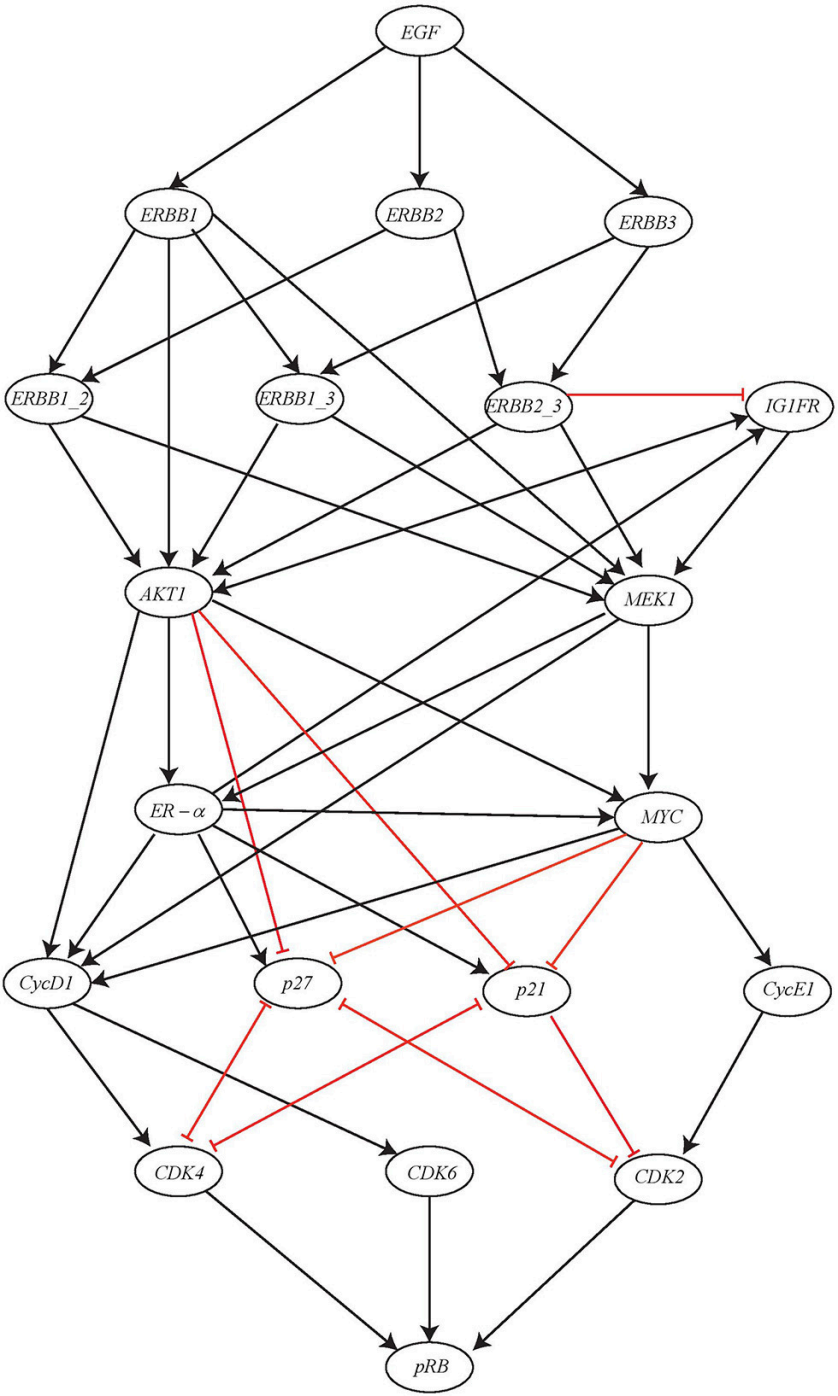
ERBB receptor-regulated G1/S transition BRN reproduced from Sahin et al. (2009).

Here we consider a comparatively large BRN of ERBB Receptor-regulated G1/S transition involved in the breast cancer and has regulations between 20 genes (Sahin et al., 2009). The BRN for this network is shown in Figure 13. The gene *pRB* is activated by this activation cascade network which subsequently controls the G1/S transition involved in cell divisions. The input of this network is the gene *EGF* which when expressed will result in potential activation of gene *pRB*.

In multicellular organisms, growth factors play a significant role in order to maintain proper growth, development and homeostasis. In regard to receptor tyrosine kinases (RTKs), the epidermal growth factor i.e., EGF family of RTKs also known as ERBB receptors is considered as hallmark of the human cancers along with process of growth, development and physiology. The ERBB family of protein functions through homodimers and hetrodimers and thus, regulates the G1/S transition during cell cycle progression in eukaryotic organisms by modulating the activity of the Cyclin D, Cyclin E/CDK complex, the c-MYC oncogene, and the p27 kinase inhibitor.

In order to maintain a continuous simulation of EGF, the initial state of *ER*−α node was set to 1. Considered to be the inhibitors of CDKs, p21 and p27 were assigned the initial state of 0. This is because the expression level of both of these inhibitors was found to be high during G0/G1 arrested cells and once cells progress through S phase, their level gets decreased because of their proteasomal degradation. Similarly, the initial state of cyclin D1 was set to 0. Now, using the above mentioned initial states, all possible state transitions were evaluated until a unique stable state was obtained that had fixed levels of all the proteins as shown in Table 3.

**Table 3:** Stable states for ERBB receptor-regulated G1/S transition obtained by PINT software (Paulevé, 2017).

**AKT1**	**CDK2**	**CDK4**	**CDK6**	**CycD1**
0	0	0	0	0
1	1	1	1	1
1	1	1	1	1
**CycE1**	**EGF**	**ERBB1**	**ERBB1_2**	**ERBB1_3**
0	0	0	0	0
1	0	0	0	0
1	1	1	1	1
**ERBB2**	**ERBB2_3**	**ERBB3**	**ERalpha**	**IGF1R**
0	0	0	0	0
0	0	0	1	1
1	1	1	1	0
**MEK1**	**MYC**	**p21**	**p27**	**pRB**
0	0	0	0	0
1	1	0	0	1
1	1	0	0	1

We apply the proposed Hybrid Process Hitting approach to this network to obtain the Hybrid Automaton for determining the time delays involved in its dynamical evolution. First we note the switch conditions and by using them we model the Hybrid Automaton. Once this Hybrid Automaton model was run in *HyTech* to compute the time delays, it was observed that the software went *Out of Memory*. This clearly showed the limitation of *HyTech* as it was unable to handle 20 Hybrid Automata at one time. The problem was overcome by utilizing the powerful reduction feature of PINT software (Paulevé, 2017) which is able to provide *Goal-oriented* model reduction through its *Cutsets* feature.

Typically a goal is the activation / inhibition of some gene in the modeled Automata Networks for BRNs. From a given initial state, the goal is reachable if a sequence of steps exists which leads to a the state in which goal is present. The sequence of states leading to the goal are known as *trace*. So *Cutsets* are sets of those local states which are included in traces leading to the goal state. Thus, the goal oriented reduction would be to apply *Cutsets* so as to remove all *traces* leading to the goal. These *Cutsets* are implemented by the knock-out of the intermediate genes present in the network.

In the ERBB network, Normal epithelial cell lines exhibit high expression levels of ERBB1 along with ERBB2 but, have low levels of ERBB3. Unlike ERBB1, ERBB2 does not show any ligand binding activity and ERBB3 tends to have defective tyrosine kinase domain. Due to which both ERBB2 and ERBB3 are unable to activate the key signaling intermediates MEK1 and AKT1. Hence, we began our activation of network through *ER*−α and eliminated all the ERBB members including, ERBB1, ERBB1-2, ERBB1-3, ERBB2, ERBB2-3, ERBB3 by applying cut-sets in PINT tool using the following command:


y = y.having(ERalpha=1) .reduce_for_goal(“AKT1=1, CDK2=1, CDK4=1, CDK6=1,
CycD1=1, CycE1=1, EGF=1, ERalpha=1, ERBB1=1, ERBB1_2=1, ERBB1_3=1, ERBB2=1,
ERBB2_3=1, ERBB3=1, IGF1R=0, MEK1=1, MYC=1, p21=0, p27=0, pRB=1”)


**Figure 14:**
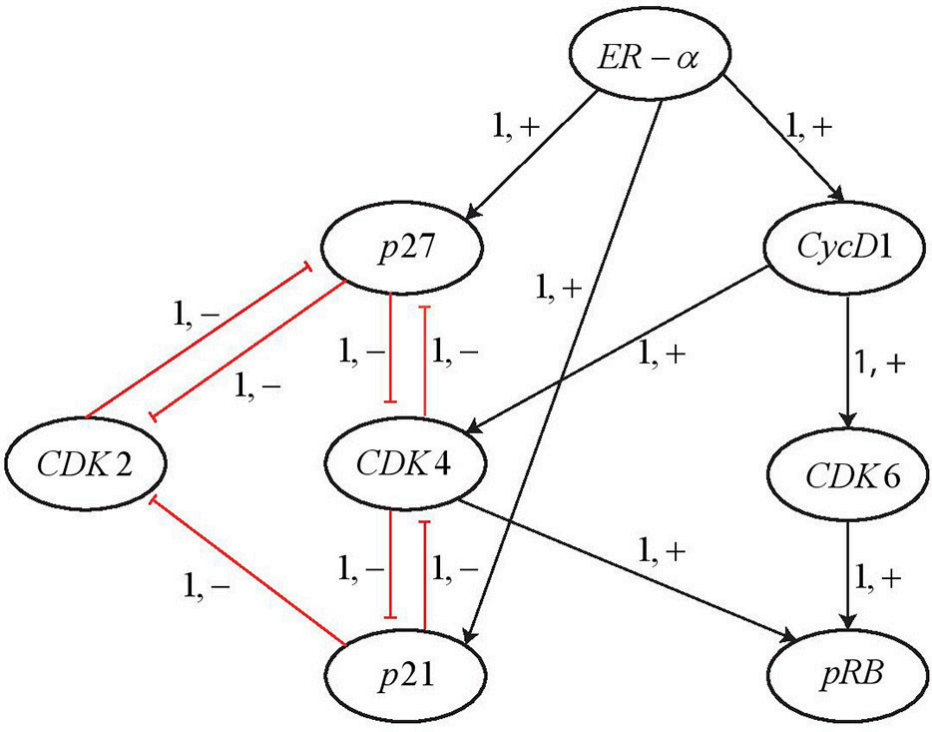
Reduced BRN for ERBB receptor-regulated G1/S transition obtained by applying the Cutsets in PINT software (Paulevé, 2017).

**Figure 15:**
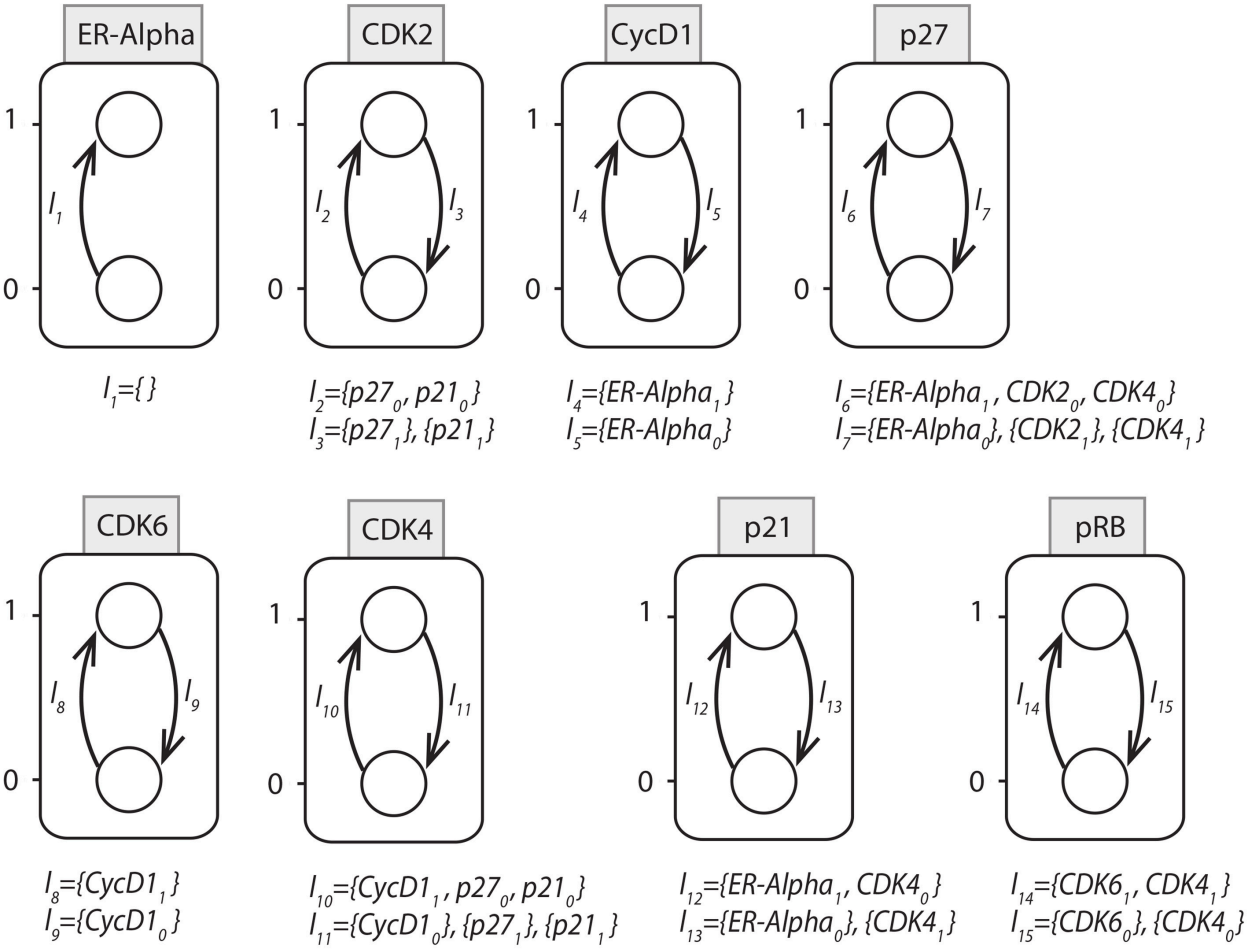
AN representation of the reduced BRN for ERBB network.

By applying the Cutsets to knock out the genes suggested by PINT, the ERBB network was reduced to 8 entities as shown in Figure 14. It can be observed that all the entities having feedback loops have been retained whereas the ones with straight forward regulations were removed. The AN representation of the reduced network is shown in Figure 15. The reduced network is then modeled as Hybrid Automata in *HyTech* and time delays are computed for the transitions between various states. The *HyTech* code and the time delays corresponding to various states of reduced ERBB network are given in the Supplementary Files 3, 4, respectively.

It is noted that *ERα* is required for the activation of *Cyclin D1*. At the pivotal point of G1/S transition, cells are dedicated to enter the S phase and results in DNA replication. This process is primarily regulated by *CycD1/CDK4/CDK6* complex that phosphorylates and thus, activates the retinoblastoma tumor suppressor protein *pRB*. For the state 〈1, 0, 1, 0, 1, 1, 0, 1〉 in which the protein *pRB* is expressed from the initial state 〈1, 0, 0, 0, 0, 0, 0, 0〉, the constraints on the time delays are obtained from the *HyTech* results as shown in Table 4.

**Table 4:** Delay Constraints obtained for the ERBB Receptor-regulated G1/S transition BRN through modeling of its corresponding system of Automata in HyTech.

**Path**	*ERα*_1_*CDK*2_0_*CycD*1_0_*p*27_0_*CDK*6_0_*CDK*4_0_*p*21_0_*pRB*_0_→
	*ERα*_1_*CDK*2_0_*CycD*1_1_*p*27_0_*CDK*6_1_*CDK*4_1_*p*21_0_*pRB*_1_
**Delay**	$d_{pRB_{1}}^{+}\geq d_{CDK4_{1}}^{+}\wedge d_{pRB_{1}}^{+}\geq d_{CDK6_{1}}^{+}\wedge d_{pRB_{1}}^{+}\geq |d_{ER\alpha_{1}}^{-}|\;\wedge$
**Constraints**	$d_{pRB_{1}}^{+}\leq d_{CDK2_{1}}^{+}\wedge d_{pRB_{1}}^{+}\geq |d_{CycD1_{1}}^{-}|\;\wedge d_{pRB_{1}}^{+}\leq d_{p27_{1}}^{+}\;\wedge$
	$d_{pRB_{1}}^{+}+|d_{CDK6_{1}}^{-}|\leq d_{CDK6_{1}}^{+}\wedge d_{pRB_{1}}^{+}+|d_{CDK4_{1}}^{-}|\leq d_{CDK4_{1}}^{+}\wedge$
	$d_{pRB_{1}}^{+}\leq d_{p21_{1}}^{+}$

*The constraints on the parametric delays for the path from the State 〈1, 0, 0, 0, 0, 0, 0, 0〉 to the State 〈1, 0, 1, 0, 1, 1, 0, 1〉, in which the protein pRB is expressed, are given*.

## 7. Conclusion and Future Work

In this paper we have proposed a new approach, *Hybrid Process Hitting*, through which the time delay information has been incorporated in the Process Hitting framework for analysis of large Biological Regulatory Networks. It has been accomplished by integrating the principles of hybrid modeling with those of Process Hitting. The proposed approach utilizes the advantage of *Process Hitting* in terms of reduction of large state graph which represents the evolution of BRN. This is achieved by considering only the permissible or possible dynamics in the state graph evolution and also by considering the combined influence in case of interactions involving multiple genes.

The feature of Cutsets is utilized for the large BRNs to knock-out some genes to achieve goal-oriented reduction through PINT software tool (Paulevé, 2017). Once the reduced BRN is obtained then the time delay variables for each gene are introduced in it which keeps track of the time taken during each step of BRN evolution. Hence, it becomes possible to obtain the constraints in terms of time delays for each gene corresponding to every evolution step of State Graph.

The time delays found through the application of Hybrid Process Hitting on the evolution of large biological networks gives us very useful information especially at the bifurcation points in the State Graph. While we can see the different paths in the State Graph through static analysis also, however, it cannot be determined which path would evolve before the others. Here, the time delay information becomes highly useful. Once we have this information, we can clearly identify the therapeutic targets to control the progress of State Graph towards desirable goals, i.e., homeostasis conditions.

It is highlighted that the proposed approach takes into consideration the concurrent evolution of all components of BRN and its modeling is realized through Hybrid Automaton. It implies that all the genes are free to evolve simultaneously as allowed by the switching conditions specified by *Process Hitting*. In this way, true dynamical behavior of BRN evolution from some given initial state to all the possible final (stable) states is obtained.

We have given the complete methodology for this approach with a running example on a toy BRN and it has also been applied to the simpler BRN of *Bacteriophage* λ and large BRN of *ERBB receptor-regulated G1/S transition* to show its tractability to large biological networks for computing the time delays associated with the transitional behavior of Network evolution.

Normal regulation of Insulin secretion related pathways pose an important aspect of pancreatic beta cells functionality. Several different types of substrates including metabolites, cytokines and neurotransmitters are involved in regulating insulin secretion through different pathways which also converge with each other at different points in this large interactome.

We plan to apply the proposed *Hybrid Process Hitting* methodology to Insulin secretion pathways considering all the known stimuli at the same time which would give us a more holistic picture of their regulation in maintaining pancreatic beta cells functional integrity. Overall, our aim is to model the collective dynamics of this insulin secretion interactome in pancreatic beta cells. This could increase our current comprehension in treating Diabetes Mellitus for which the first line of treatment is the use of oral anti-diabetic drugs which mainly target these pathways.

## Author Contributions

IS and JA conceived and developed the methodology, conducted the experiments and writing of manuscript. All the authors took part in discussions, analysis and layout of results, and reviewed the manuscript.

### Conflict of Interest Statement

The authors declare that the research was conducted in the absence of any commercial or financial relationships that could be construed as a potential conflict of interest.
