# Supplementary material for: Incorporating Time Delays in Process Hitting Framework for Dynamical Modeling of Large Biological Regulatory Networks
Source: Front Physiol. 2019 Feb 15;10:90. doi: 10.3389/fphys.2019.00090 (PMC6385622; doi:10.3389/fphys.2019.00090)
Supplement: Supplementary file 2 [file Data_Sheet_2.PDF]

Glossary of Mathematical Definitions

**Notation**	**Description**
IG	Interaction Graph
N	Set of all Nodes
E	Set of all Edges
t	Qualitative threshold level required for interaction
l_p	Highest threshold level of gene p
s	Type of interaction, '+' for activation and '-' for inhibition
LEV_p^+	Effective Levels at and above which p will activate
LEV_p^-	Effective Levels at and below which p will inhibit
AN	Automata Network
Σ	Finite set of Automata
L	Finite set of global states
L_p	Finite set of local states
**LS**	Set of all the local states
$\mathcal{H}$	Set of all local transitions in the network
$\mathcal{H}_p$	Set of local transitions on automaton p
θ	Step; Subset of local transitions
$^\bullet\theta$	Origin of Step
ℓ	Condition of Step
$\theta^\bullet$	Destination of Step
π	Trace; Sequence of successive steps
l_T	Goal State
$Cond_{ACT}$	Regulation Condition
$Cond_{INH}$	Inhibition Condition
μ	Hit part of a Process Hit
$\bigwedge$	Switch Conditions for Gene Regulation
h_p	Clock Variable
d_p^+	Production Delay
d_p^-	Degradation Delay
φ	Clock Constraints
B	Parametric Biological Linear Hybrid Automaton (Bio-LHA)
L	Finite set of locations
l_0	Initial location
D	Finite set of parameters (delays)
X	Finite set of real-valued variable (clocks)
E	Finite set of Edges
g	Guard on the clock constraints
Inv	Invariant for any location
Dif	Rate of evolution
P	Parameter
γ	Valuation for the parameters
ν	Value of clock in a location
B_N	Network of Hybrid Automaton
